# Circulating brain-derived neurotrophic factor as a potential biomarker in stroke: a systematic review and meta-analysis

**DOI:** 10.1186/s12967-022-03312-y

**Published:** 2022-03-14

**Authors:** Helia Mojtabavi, Zoha Shaka, Sara Momtazmanesh, Atra Ajdari, Nima Rezaei

**Affiliations:** 1 School of Medicine, Tehran University of Medical Sciences, Tehran, Iran; 2 Systematic Review and Meta-Analysis Expert Group (SRMEG), Universal Scientific Education and Research Network (USERN), Tehran, Iran; 3 Metacognition Interest Group (MCIG), Universal Scientific Education and Research Network (USERN), Tehran, Iran; 4 Research Center for Immunodeficiency, Children’s Medical Center Hospital, Tehran University of Medical Sciences, Dr. Qarib St, Keshavarz Blvd, 14194 Tehran, Iran; 5 Department of Immunology, School of Medicine, Tehran University of Medical Sciences, Tehran, Iran

**Keywords:** Brain-derived neurotrophic factor, Meta-analysis, Neuroplasticity, Stroke, Systematic review, Exercise, Depression

## Abstract

**Background:**

Stroke, an acute cerebrovascular event, is a leading cause of disability, placing a significant psycho-socioeconomic burden worldwide. The adaptation and reorganization process following any neuronal damage is regarded as neuroplasticity. Among many factors believed to attribute to this process, Brain-derived Neurotrophic Factor (BDNF) is a neurotrophin coordinating neuroplasticity after various neurological disorders such as stroke.

**Methods:**

We conducted a systematic search in the main electronic medical databases in January 2021. Primarily we want to compare BDNF levels between patients with stroke and healthy controls (HC). Additional aims included investigation of (1) longitudinal changes in the BDNF levels post-stroke, (2) effects of physical training, (3) repeated transcranial magnetic stimulation (rTMS), and presence of depression on BDNF levels in patients with stroke.

**Results:**

Among 6243 reviewed records from PubMed, Web of Science, and Scopus, 62 studies were eligible for inclusion in our systematic review. Subjects with stroke, n = 1856, showed lower BDNF levels compared to HC, n = 1191 (SMD [95%CI] = − 1.04 [− 1.49 to − 0.58]). No significant difference was detected in the level of BDNF through time points past stroke. BDNF levels were lower in the patients with depression compared to non-depressed subjects (SMD [95%CI] = − 0.60 [− 1.10 to − 0.10]). Physical training had an immediate positive effect on the BDNF levels and not statistically significant effect in the long term; SMD [95%CI] = 0.49 [0.09 to 0.88]) and SMD [95%CI] = 0.02 [− 0.43 to 0.47]). Lastly, rTMS showed no effect on the level of BDNF with 0.00 SMD.

**Conclusions:**

Our study confirms that stroke significantly decreases the level of BDNF in various domains such as cognition, affect, and motor function. As BDNF is the major representative of neuroplasticity within nervous system, it is believed that stroke has a significant impact on the CNS regeneration, which is permanent if left untreated. This effect is intensified with coexisting conditions such as depression which further decrease the BDNF level but the net impact yet needs to be discovered. We also conclude that exercise and some interventions such as different medications could effectively reverse the damage but further studies are crucial to reach the exact modality and dosage for their optimal effect.

**Supplementary Information:**

The online version contains supplementary material available at 10.1186/s12967-022-03312-y.

## Introduction

Beyond 13 million people are affected by stroke annually, which is among the leading causes of incapacity and mortality, placing a significant psycho-socio economic burden worldwide [1]. Through the advent of medical techniques, mortality rates following stroke have dropped remarkably, leading to higher disability-adjusted life years (DALY) [2]. Approximately 26% of patients with stroke (PwS) will face levels of dependency in their daily activities up to six months after the stroke onset [3]. However, the motor function is not the mere focus of disabilities among PwS; patients’ cognitive profile and affect are also altered by stroke [4, 5].

Acute stroke is referred to the acute onset of focal neurological findings in a vascular territory. There are two main types of stroke, following a vascular occlusion resulting in an infarct, regarded as ischemic stroke (85%), or caused by a sudden burst of a blood vessel, regarded as hemorrhagic stroke (15%). In response to this cerebrovascular event, CNS uses an ability to adapt and reorganize the damage known as “Neuroplasticity”. A group of signaling molecules known as neurotrophins play essential roles in synaptic plasticity, neurogenesis and survival regulations in neural cells [6–8]. In particular, many studies have investigated Brain-Derived Neurotrophic Factor (BDNF) as a member of the neurotrophins family for its role in the nervous system [9–11]. Extensive evidence demonstrated some associations between BDNF level and various neurological disorders; for instance, low concentrations of BDNF has been reported in neurodegenerative and neuropsychiatric disorders such as Alzheimer’s disease, Parkinson’s disease, depression, autism spectrum disorders, and post-traumatic stress disorder [9–12].

Keen interest has been raised to find the relationship between the altered level of BDNF and stroke; studies hypothesize that BDNF level is correlated with the size of the stroke legion, cognitive profile, and functional outcome [13–15]. Based on the substantial impact of stroke, a systematic review and meta-analysis are essential to address the precise association of circulating BDNF in PwS in various conditions.

## Materials and method

### Literature search and selection criteria

We conducted a comprehensive systematic search by using the keywords “stroke” OR “ischemic stroke” OR “hemorrhagic stroke” OR “Cerebrovascular Accident” OR “Brain Vascular Accident” AND “Brain-Derived Neurotrophic Factor” OR “BDNF” on PubMed, Web of Science and Scopus in January 2021. Original articles were included by the following criteria; (1) a study measuring serum or plasma levels of BDNF in PwS. Studies (a) with animal subjects, (b) review or meta-analysis studies, (c) genetic investigation were excluded from the study (d) using other measuring methods rather than ELISA, like western blot, mass spectrometry or immunohistochemistry.

**Fig. 1:**
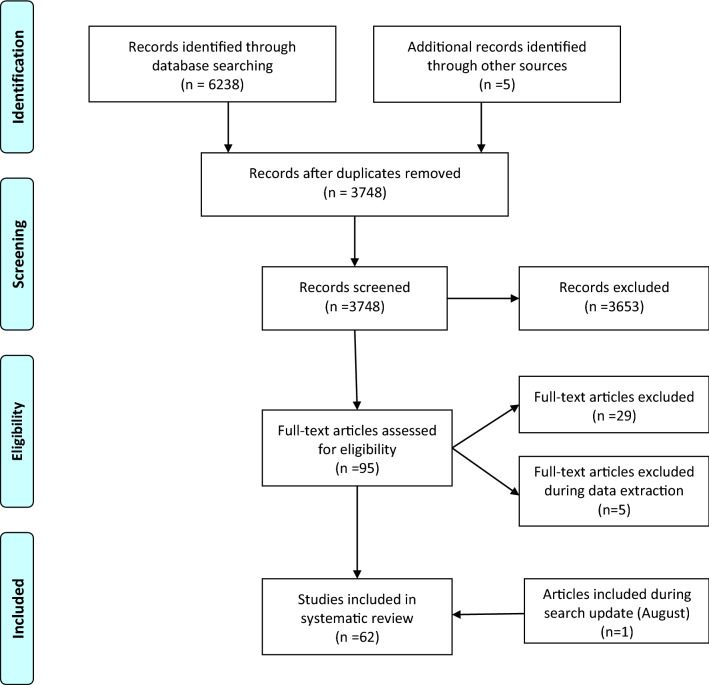
PRISMA flow diagram

Additionally, the reference list and citations of all included articles were checked to find potential eligible articles, resulting in 5 eligible articles. We updated our search, using the exact keywords, on August 7th 2021, which resulted in the inclusion of one study [16]. Finally, a total number of 62 articles were entered into our systematic review (Fig. 1).

### Data extraction

The following data were extracted from each included publication; the name of the first author, year of publication, location of study (country), type of specimen (serum, plasma or CSF), number of subjects in each study group, demographic characteristics (i.e., age and gender) of both groups, stroke type (ischemic or hemorrhagic), stroke site, patients’ score for National Institutes of Health Stroke Scale (NIHSS), post-stroke duration at the time of sample collection, mean and standard deviation (SD) of the peripheral BDNF levels, and the assay used for BDNF measurement.

Two independent researchers extracted data from each manuscript, and the mismatch was discussed and resolved afterwards. In the case of figure illustration, the data were interpreted from the figure using an online interpreter [17]. The corresponding/first authors were contacted and invited to share their results if the two prior approaches were unsuccessful. An excel spreadsheet was made from the relevant data and is available on request. Characteristics of the included studies is illustrated in Table 1.

**Table 1:** Characteristics of the included studies

Study ID	Sample	CVA type	Groups				NIHSS mean
			characteristic	N	M/F	Age	
Algin 2019, Turkey	Serum	Ischemic	stroke	75	34/43	73.22	10.88
			Healthy control	28	8/20	69.14	
Anjum 2020, Pakistan	Serum	Hem/Isch	CIMT	20	NA	NA	NA
			Physiotherapy	20	NA	NA	
Asadollahi 2019, Iran	Serum	Ischemic	Saffron—treated	19	11/8	70.16	T1: 11.30 T2: 9.15
			Standard treatment	20	1/4	72.25	
Bastawy 2019, Egypt	Serum	Hem/Isch	Graded exercise test	35	23/15	65.2	4.29
Bembenk 2020, Poland (full text not found)	NA	NA	Chronological study baseline, 4 weeks, 3 month	61	NA	NA	NA
Billinger 2018, USA	Serum	Hem/Isch	Chronological study baseline, day7	13	7/6	62.08	NA
Bintang 2020, Indonesia	Serum	ischemic	rTMS	14	6/8	54.50	NA
			control	13	8/5	62.15	
Boyne 2019, USA	NA	Hem/Isch	Graded exercise test	16	9/7	57.4	NA
Casas 2017, Argentina	Plasma	Ischemic	Acute stroke	40	20/20	77.5	12.25
			Healthy control	20	10/10	73.5	
Chan 2015, Australia	Serum	Ischemic	stroke	75	44/31	69	3.5
			Healthy control	56	29/27	43	
Chang 2018, Korea	Serum	Hem/Isch	Chronological study baseline, day7, week2	38	23/15	62.9	7.5
Chaturvedi 2020, India	Serum	Hem/Isch	PNF	208	126/82	55.29	9.36
Chen 2018, China	Serum	NA	Acute stroke	30	17/13	65.9	NA
			Healthy control	30	16/14	66.2	
Cichon 2018, Poland	Plasma	Ischemic	ELF-EMF group	25	15/10	48.0	5.16
			non ELF-EMF group	23	11/12	44.8	
de Morais 2018, Brazil	Serum	Ischemic	mild intensity walking	10	5/5	58	NA
Di Lazzaro 2017, Italy	Serum	Ischemic	Single group	10	5/5	67.9	7.2
El-Tamawy 2014 Egypt	Serum	NA	PT	15	10/5	49.67	NA
			Aerobic exercise + PT	15	11/4	48.4	
Greisenegger 2015, UK	Serum	TIA	Survivors	568	282/286	68	0
			Non survivors	361	178/183	82	
Han 2020, China	Serum	Hem/Isch	PSD	61	35/26	67.89	NA
			PSA	40	22/18	67.23	
			Without any	61	37/ 24	67.97	
Hassan 2018, Neigeria	Serum	NA	Stroke	47	27/20	55	NA
			Healthy control	35	18/17	50	
Hsu 2020, Taiwan	Serum	NA	HIIT group	10	8\2	58.5	NA
			MICT group	13	12\1	53.1	
Hidayat 2016, Indonesia	Serum	Ischemic	Stoke with different stroke onset	17	NA	61.75	NA
Hutanu 2020, Romania	Serum	Ischemic	Stroke	114	65/49	71.7	NA
			Healthy control	40	23/17	67.9	
Jiménez 2008, Spain	Serum	Ischemic	PSD	109	79/30	69	3
			Non-PSD	25	13/12	77	
Karakulova 2018, Russia	Serum	Ischemic	IV-Cytoflavin with basal therapy	25	NA	NA	T1: 6.5 T2: 5.53
			standard treatment	27	NA	NA	
Kim 2019, Korea	NA	Hem/Isch	LI-aerobic exercise plus dual-task training	9	4/5	59.77	NA
			MI- aerobic exercise plus dual-task training	9	4/5	58.22	
			HI- aerobic exercise plus dual-task training	9	5/4	57.55	
King 2019, Canada	Serum	Hem/Isch	Exercise	35	NA	65.2	4.29
Koroleva 2020, Russia	Serum	Ischemic	Stroke	50	28/22	62.5	NA
			Healthy control	50	29/21	60.5	
Kotlega 2020, Poland	Plasma	Ischemic	Single group	73	33/40	60.7	NA
Kozak 2019, Turkey	Serum	Ischemic	AIS	36	19/17	65.25	6.96
			AIS and DM	17	8/9	67.47	
Kozak 2016, Turkey	Serum	Ischemic	AIS	49	24/25	64.63	T1: 6.88 T2: 3.43
			AIS and delirium	11	5/6	72.91	
Lasek-Bal 2015, Poland	Serum	Ischemic	Single group	87	45/42	71.7	NA
Lasek-Bal 2019, Poland	NA	Ischemic	Single group	138	63/75	73.11	3
Levchuk 2019, Russia	Serum	Ischemic	Stroke	68	NA	NA	NA
			Health control	20	NA	NA	
Li 2014, China	Serum	Ischemic	with Depression	59	24/35	72.8	5.81
			without depression	157	93/64	63.6	
Lopez Cancio 2017, Spain	Serum	Ischemic	Chronological study baseline, day7, month3	83	48/35	69.6	16.68
Lu 2015, China	Plasma	Hem/Isch	With dysmnesia	40	25\15	44.9	NA
			Healthy control	10	6/4	42.4	
Luo 2019, China/USA	Serum	Hem/Isch	Low FIM on admission	174	98/76	68.9	NA
			High FIM on admission	174	98/76	66.5	NA
Mirowska-Guzel 2013, Poland	Serum	Ischemic	Low-frequency rTMS	46	29/17	62.09	NA
Mourao 2019, Brazil	Plasma	Ischemic	Chronological study 72 h, at discharge	50	28/22	65.5	T0:8.04 T1: 7.86 yT2: 5.89
Niimi 2016, Japan	Serum	Hem/Isch	rTMS	62	41/21	62.3	NA
			Routine treatment	33	17/16	66.2	
Ortega 2019, Spain	Plasma	Ischemic	Non-PSH	95	69.7	46/49	4.54
			PSH	79	72.1	25/54	
Pascotini. 2018, Brazil	Plasma	Ischemic	stroke	44	27\13	63	NA
			Healthy control	44	22/18	56	
Pedard 2018, France	Serum	Ischemic	Chronological study day1, at discharge	40	20/20	76.81	T0: 7.34 T1: 3.53 T2: 1.34
Prodjohardjono 2020, Indonesia	Serum	Ischemic	Chronological study day5, day 30	68	60.97	36\32	NA
Qiao 2017, China	Serum	Ischemic	Stroke	270	144\126	65	7
			Healthy control	100	53/47	65	
Rodier 2015, France	Serum	Hem/Isch	rt-PA-treated Patients	24	8/6	69.13	NA
			Non-treated Patients	14	5/9	74.71	
Roslavtceva 2020, Russia	Plasma	Ischemic	Chronological study baseline, day7, week3	56	21/35	63	NA
Ryan 2019, USA	Plasma	Chronic stroke	Conventional rehabilitation therapy	16	NA	62	NA
Santos 2016, Brazil	Serum	NA	Stroke	17	NA	62.71	NA
			Healthy control	17	NA	61.88	
Siotto 2017, Italy	Serum	Hem/Isch	Sub-acute patients	19	10/9	67.5	NA
			Chronic patients	31	16/15	72.1	
Silva Mariana 2017, Brazil	Serum	Hem/Isch	Low-intensity walk	15	9/6	60.8	NA
Sobrino 2020, Spain	Serum	Ischemic	Favorable outcome	351	214/137	66.9	11.66
			Unfavorable outcome	201	128/73	69.6	
Stanne 2016, UK	Serum	Ischemic	Stroke	491	NA	NA	NA
			Healthy control	513	NA	NA	
Syafrita 2020, Indonesia	Serum	Ischemic	With depression	36	18/18	59.67	NA
			Without depression	38	19/17	59.64	
Wang 2019, China	Serum	Ischemic	stroke	40	20/18	62.5	10
			Healthy control	40	21/19	62.5	
Wang 2017, China	Serum	Ischemic	Stroke	204	110/94	64	6
			Healthy control	100	NA	NA	
Wang 2021, China	Serum	Hem/Isch	Rehabilitation	50	65.3	32/18	NA
			Routine treatment	50	66.5	30/20	
			Healthy control	50	64.2	26/24	
Widodo 2016, Indonesia	Serum	Ischemic	7–30 days post stroke	19	NA	58	NA
			> 30 days post stroke	27	NA	56	
Yang 2011, China	Serum	Ischemic	PSD	37	18/19	68.95	T0: 4.48 T1: 2.48
			Non PSD	63	37/26	68.43	
			Healthy control	50	22/28	65.12	
Zhang 2017, China	Serum	ischemic	Atorvastatin	37	21/16	65.11	8.11
			Control group	38	23/15	63.34	
Zhou 2011, China	Serum	ischemic	PSD	35	19/16	61.7	5.75
			Non-PSD	58	34/21	63.5	
			Healthy controls	30	NA	NA	

CIMT: constraint- induced movement therapy, PT: physiotherapy, rTMS: Repetitive transcranial magnetic stimulation, PNF: proprioceptive neuromuscular facilitation exercises, ELF-EMF: extremely low-frequency electromagnetic field therapy, HIIT: high-intensity interval training, MICT: moderate-intensity continuous training (MICT), AIS: acute ischemic stroke, DM: Diabetes mellitus, FIM: functional independence measure, PSH: post stroke hyperglycemia, rtPA: The recombinant form of tissue plasminogen activator, PSD: Post stroke Depression, PSA: Post stroke Anxiety, LI, MI, HI: low, medium and high intensity

### Quality assessment

**Fig. 2:**
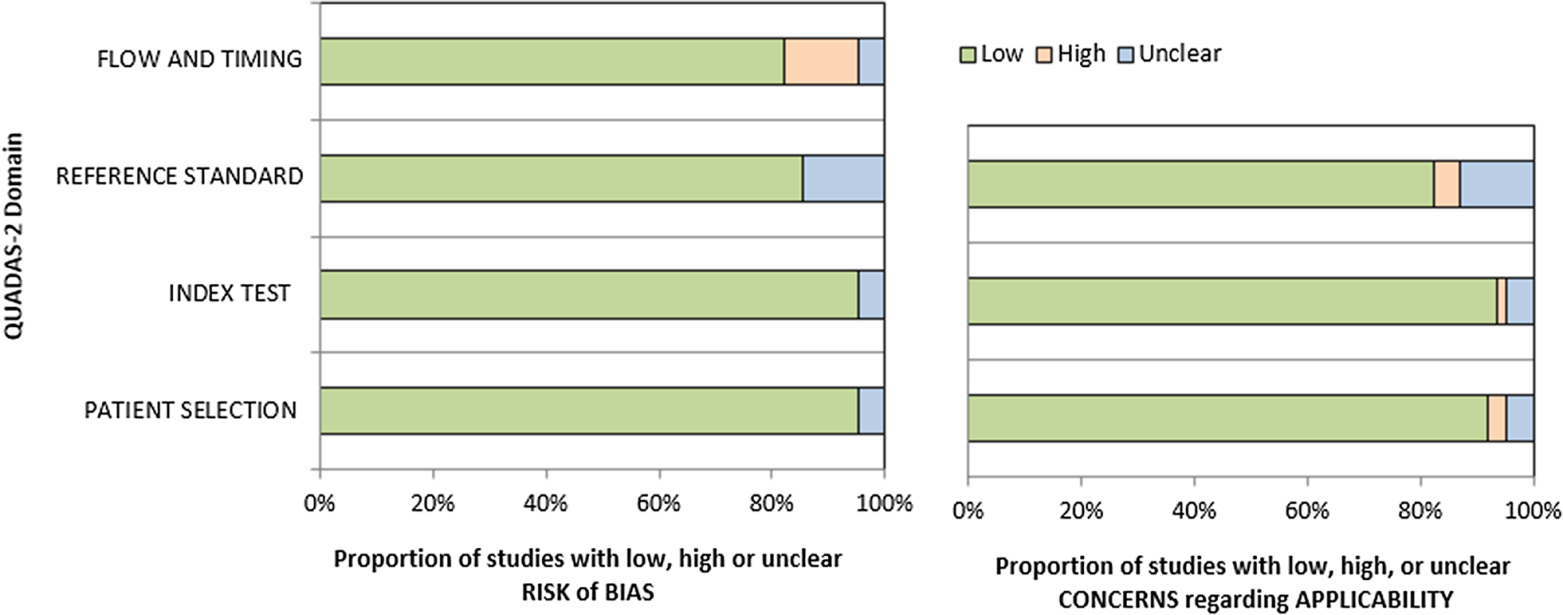
Proportion of risk of bias assessment by each domain

Two authors performed the methodological quality assessment independently to evaluate each included study's risk of bias and applicability by QUADAS-2 tool [18]. This tool is designed to assess the risk of bias in four main domains, including patient selection, index test, reference standard and, flow and timing. Concerns regarding the applicability were also apprised in the first three domains. In each domain, there are three possible rankings; studies categorized as “high” represent the lowest quality with the highest risk of bias and concerns, while studies categorized as “low” indicate the highest quality with the lowest risk of bias and considerations. The “unclear” category was used for studies that reported insufficient data in each domain. Any disagreement was resolved by discussion or consultation with another author (Table 2 and Fig. 2).

**Table 2:** QUADAS

Study ID	Risk of bias				Applicability concerns			
	Patient selection	Index text	Reference standard	Flow and timing	Patient selection	Index text	Reference standard	Score (0–7)
Algin 2019	Low	Low	Low	Low	Low	Low	Low	**7**
Anjum 2020	Low	Low	Low	Low	Low	Low	Low	**7**
Asadollahi 2019	Low	Low	Low	Low	Low	Low	Low	**7**
Bastawy 2019	Low	Low	Low	Low	Low	Low	Low	**7**
Bembenek 2020 (full text not found)	Unclear	Unclear	Unclear	Unclear	Unclear	Unclear	Unclear	**-**
Billinger 2017	Low	Low	Low	High	Low	Low	Low	**6**
Bintang 2020	Low	Low	Low	Low	Low	Low	Low	**7**
Boyne 2018	Low	Low	Low	Low	Low	Low	Low	**7**
Casas 2017	Low	Low	Low	Low	Low	Low	Low	**7**
Chan 2015	Low	Low	Low	Low	Low	Low	Low	**7**
Chang 2018	Low	Low	Unclear	High	Low	Low	Low	**5**
Chaturvedi 2020	Low	Low	Low	Low	Low	Low	Low	**7**
Chen 2018	Low	Low	Low	Low	Low	Low	Low	**7**
Cichon 2018	Low	Low	Low	Low	Low	Low	Low	**7**
de Morais 2018	Low	Low	Low	Low	Low	Low	Low	**7**
Di Lazzaro 2007	Low	Low	Low	Low	Low	Low	Low	**7**
El-Tamawy 2014	Low	Low	Unclear	Low	Low	Low	Unclear	**5**
Greisenegger 2015	Low	Low	Low	High	Low	Low	High	**5**
Han 2020	Low	Low	Low	Low	Low	Low	Low	**7**
Hassan 2018	Low	Low	Low	Low	Low	Low	Low	**7**
Hsu 2020	Low	Low	Low	Low	Low	Low	Low	**7**
Hidayat 2016	Low	Low	Low	Low	Low	Low	Low	**7**
Hutanu 2020	Low	Low	Low	Low	Low	Low	Low	**7**
Jimenez 2008 (full text in Spanish)	Unclear	Unclear	Unclear	Unclear	Unclear	Unclear	Unclear	**-**
Karakulova 2018	Low	Low	Low	Low	Low	Low	Low	**7**
Kim 2019	Low	Low	Low	Low	Low	Low	Low	**7**
King 2019	Low	Low	Unclear	High	Low	Low	Unclear	**4**
Koroleva 2020	Low	Low	Low	Low	Low	Low	Low	**7**
Kotlega 2020	Low	Low	Low	Low	Low	Low	Low	**7**
Kozak 2019	Low	Low	Low	Low	High	Low	Low	**6**
Kozak 2016	Low	Low	Low	Low	Low	Low	Low	**7**
Lasek-Bal 2015	Low	Low	Low	Low	Low	Low	Low	**7**
Lasek-Bal 2019	Low	Low	Low	Low	Low	Low	Low	**7**
Levchuk 2019	Low	Unclear	Low	Low	Low	Unclear	Low	**5**
Li 2014	Low	Low	Low	High	Low	Low	Low	**6**
Lopez-Cancio 2017	Low	Low	Low	Low	Low	Low	Low	**7**
Lu 2015	Low	Low	Low	Low	Low	Low	Low	**7**
Luo 2019	Low	Low	Low	Low	Low	Low	Low	**7**
Mirowska-Guzel 2013	Low	Low	Low	Low	High	Low	Low	**6**
Mourao 2019	Low	Low	Low	Low	Low	Low	Low	**7**
Niimi 2016	Low	Low	Low	Low	Low	Low	Low	**7**
Ortega 2019	Low	Low	Low	Low	Low	Low	Low	**7**
Pascotini 2018	Low	Low	Low	Low	Low	Low	Low	**7**
Pedard 2018	Low	Low	Unclear	Low	Low	Low	Unclear	**5**
Prodjohardjono 2020	Low	Low	Low	Low	Low	Low	Low	**7**
Qiao 2017	Low	Low	Low	Low	Low	Low	Low	**7**
Rodier 2015	Low	Low	Low	Low	Low	Low	Low	**7**
Roslavtceva 2020	Low	Low	Low	Low	Low	Low	Low	**7**
Ryan 2019	Low	Low	Unclear	Low	Low	Low	Unclear	**5**
Santos 2016	Low	Low	Unclear	High	Low	Low	Unclear	**4**
Siotto 2017	Low	Low	Low	High	Low	Low	High	**5**
Silva Mariana 2017	Low	Low	Low	Low	Low	Low	Low	**7**
Sobrino 2020	Low	Low	Low	High	Low	Low	Low	**6**
Stanne 2016	Unclear	Low	Unclear	Low	Unclear	High	Unclear	**2**
Syafrita 2020	Low	Low	Low	Low	Low	Low	Low	**7**
Wang 2021	Low	Low	Low	Low	Low	Low	Low	**7**
Wang 2019	Low	Low	Low	Unclear	Low	Low	Low	**6**
Wang 2017	Low	Low	Low	Low	Low	Low	Low	**7**
Widodo 2016	Low	Low	Low	Low	Low	Low	Low	**7**
Yang 2011	Low	Low	Low	Low	Low	Low	Low	**7**
Zhang 2017	Low	Low	Low	Low	Low	Low	Low	**7**
Zhou 2011	Low	Low	Low	Low	Low	Low	High	**6**

### Quantitative analysis

All statistical analyses were performed using “meta” (version 4.17–0), “metafor” (version 2.4–0), and “dmetar” (version 0.0–9) packages, R (R Core Team (2020). R: A language and environment for statistical computing. R Foundation for Statistical Computing, Vienna, Austria) and STATA. A p-value of < 0.05 was considered statistically significant. 

#### Meta-analysis

We reported standardized mean difference (SMD) (Hedge's g) and 95% confidence interval (CI) for each between-group comparison. The SMD of $\le$ 0.2, 0.2–0.8, and 0.8 $\le$ represented small, moderate, and large effect sizes, respectively.

The Cochran’s Q test and the I^2^-index were used to investigate heterogeneity between studies. The I^2^-indices of $\le$ 25%, 26–75%, and 75% ≤ represented low, moderate, and high degrees of heterogeneity, respectively [19]. Fixed effects models were applied if the results were homogeneous (I^2^ < 40% and *p-value* > 0.05), and random effect models according to the DerSimonian and Laird method [20] were used if these results were heterogeneous (I^2^ ≥ 40% or *p-value* ≤ 0.05) [21].

Our primary aim was to compare BDNF levels between PwS and healthy controls (HC). Additionally, we aimed to address (1) the longitudinal changes, (2) the effects of physical training, (3) repeated transcranial magnetic stimulation (rTMS), and (4) clinical diagnosis of post-stroke depression (PSD) on the circulating BDNF levels in PwS.

Within the groups undergoing additional analysis, some concerns existed in the physical training group. First, various training modalities were implemented, ranging from regular post-stroke physiotherapy sessions to different exercise protocols in addition to the variation in each session’s duration. Lastly, sample collection time varied between studies immediately after the session completion to weeks and months after the whole program was terminated.

To overcome this diversity and reach a reasonable grouping modality with maximum homogeneity, this is besides the heterogeneity and sensitivity analysis, we divided studies into two primary groups according to the time of sample collection, those who collected samples (1) immediately after the exercise (within 20 min to 24 h) and (2) with a time delay after the training was completed (within two weeks to a couple of months). The variety of the training protocols was also an interesting point throughout the studies. Thus we conducted a subgroup analysis between routine physiotherapy protocol and other exercise protocols. Although there were several specifications for the exercising routines like High-Intensity interval training (HIIT), we could not devote a specific subgroup for each due to the inadequate number of observations.

It is noteworthy that some of the included studies had applied two or more physical interventions, such as routine physiotherapy, aerobic exercise, and non-aerobic exercise, into different study groups, leading to more observational groups being included from the study. Similarly, many studies have reported both immediate and delayed levels so we could extract more than one observational groups from a single study. This clearly describes repeated study names in our meta-analysis results. Table 3 illustrates a description of the included studies in our physical training group in detail.

**Table 3:** PT protocols

Study ID	Intervention modality	Number of sessions	Session duration	Time of sample collection
Anjum 2020	Constraint-induced movement therapy (CIMT) using motion capture video gaming technology (Nintendo Wii)	16	20 min	Before, After each session
Bastawy 2019	Incremental maximal aerobic exercise test on either a supported treadmill or on a total body recumbent stepper	NA	12 ± 6 min	Before, After each session
Chang 2018	Occupational and Physical therapy	10	60 and 120 min	Before, one week and, 2 weeks after the program
Chaturvedi 2020	Proprioceptive neuromuscular facilitation exercise (PNF), specific exercises designed for neck, scapula, pelvis, and trunk	20	30 min	Before, after each session
de Morais 2018	Beginning the session by walking on the ground to reach the target heart rate with progressive intensity, and then gradually slowed down. After training, participants performed stretching as a cool down activity	2	60 min	Before, after each session
El-Tamawy 2014	Physiotherapy program	24	25–30 min	Before, after the program
	Aerobic exercise on a Bicycle ergometer (Monark Rehab trainer model 88 E)	24	40–45 min	
Hsu 2020	MICT (moderate-intensity continuous training) or HIIT (high-intensity interval training) on a bicycle ergometer	36	36 min	Before, after each session
Koroleva 2020	Augmented reality (AR)-based training to visualize sensory stimuli	10	20 min	NA
Kim 2019	Treadmill aerobic exercise was performed at low intensity (gourp1), moderate intensity (group2) and high intensity (group3)	1	26–46 min	7 days before, and 1 day after training
	Dual-task training on treadmill was performed additionally for all the 3 groups	30	NA	
King 2019	Incremental maximal aerobic exercise performed on either a body weight supported treadmill or on a total body recumbent stepper	NA	12 min 46 s ± 6 min 4 s	Before, after each session
Levchuk 2019	Not declared			
Mirowska-Guzel 2013	Conventional individual physiotherapy	15	45 min	before, after the first 6 h, and 3 weeks
Niimi 2016	Shaping techniques (reaching forward to move a cup from one place to another, wiping the surface of the table with a towel, picking up a hairbrush and combing hair, writing letters with a pencil)and repetitive task practice ( turning over cards, squeezing clay, gripping a small ball, and pinching small coins)	28	60 min	Before, immediately after and after 14 days after
Ryan 2019	Resistive training unilateral repetitions on the leg press, leg extension, and leg curl machines (Keiser; pneumatic resistance, Fresno, CA)	36	40 min	Before, after each session
	Aerobic exercise training on treadmill	36	40 min	
Silva Mariana 2017	Low-intensity walk on treadmill	Single session	30 min	Before, after the session
Wang 2021	1. Rehabilitation training for attention 2. Training for thinking ability 3. Rehabilitation training for memory 4. Rehabilitation training for orientation ability 5. Rehabilitation training for perceptual ability	56	30 min	Before each session

We had no concerns about the categorization of the other additional group analysis.

#### Regression and sensitivity analysis

To further assess causes of heterogeneity, we conducted separate regression of mean age and mean NIHSS2 on SMD whenever the required data was available for a number ten or more studies. Moreover, for the meta-analyses including ten or more studies that had significant heterogeneity, we conducted sensitivity analysis to identify influential articles. We performed Leave-One-Out analysis by omitting one study each time and recalculating the effect size. We also applied the diagnostic procedure developed by Viechtbauer and Cheung [22] to detect influential cases and Baujat Plot [23] to investigate each study's effect on the effect size and heterogeneity.

#### Meta bias

We assessed publication bias by visual inspection of the degree of funnel plot asymmetry. Egger bias test [24] and Begg-Mazumdar Kendall's tau [25] were also performed to confirm the visual perception from the funnel plot objectively. A p-value < 0.1 was considered as evidence of publication bias.

## Results

Our initial database search revealed a total number of 6243 citations. We removed 2495 duplicate citations, and 3653 additional citations were excluded through title and abstract screening. We retrieved 95 articles for full-text screening, from which 29 studies were excluded for the following reasons: animal sample size, review article, duplicated articles from one same study, in vitro study, and reporting one single measure in one single study group at only one-time point. An additional number of 5 articles were removed during the data extraction because of: insufficient data despite author contact [22, 26], reporting BNGF instead of BDNF [23], unclear data from an extensive cohort study (Framingham [3]), reporting peripheral BDNF in association with another biomarker instead of pure measures [27].

Finally, a total number of 62 articles were entered into our systematic review. We could not enter 16 articles to any meta-analysis group for the following reasons; providing delta BDNF instead of exact measures [28], reporting peripheral BDNF as a proportion to the control group [29], the full text was not found. However the abstract was sufficient to extract the descriptive data [30], unsatisfactory data and irresponsive author (missing SD despite reporting the mean) [31], single study group with no comparison or specific grouping which did not fall into our meta-analysis categorizations [14, 32–42].

### Serum BDNF levels in PwS vs. HC

**Fig. 3:**
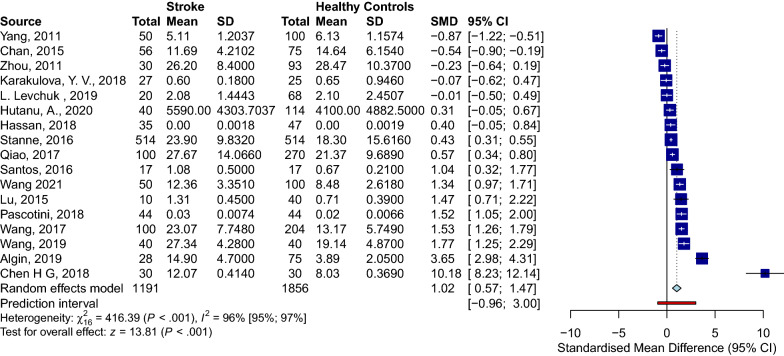
Forest HC stroke

**Fig. 4:**
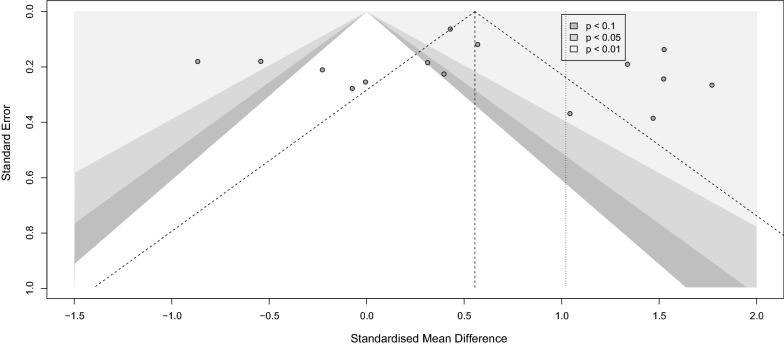
Funnel HC stroke

Seventeen studies assessed the differences between serum BDNF levels in PwS (n = 1191) and HC (n = 1856) [13, 16, 43–57]. PwS had significantly lower serum BDNF levels than HC (SMD [95%CI] = − 1.02 [− 1.47 to − 0.57], *p-value* < 0.001, I^2^ = 96%, *p-*value < 0.001) (Fig. 3). No publication bias was detected (Fig. 4). Sensitivity analysis showed that Align et al. [58] and Chan et al. [54] were influential (Additional file 1: Fig. S1). After excluding these studies, the meta-analysis of the remaining fifteen studies showed significantly lower serum BDNF levels in PwS than HC as well (− 0.92 [− 1.35 to − 0.50], *p-*value < 0.001, I^2^ = 96%, *p-*value < 0.001). To further assess sources of heterogeneity, we performed meta-regression. The age partially explained the high heterogeneity (correlation coefficient = − 0.11, R^2^ = 62.81%, *p-*value = 0.000).

### Longitudinal investigation within stroke population

To address the pattern of serum BDNF level through the time of stroke onset, 23 studies longitudinally assessed their samples. A total number of 3678 patients were included in 39 observations. Time points for sample collection varied extremely between the studies. After the precise data extraction for the time of sample collection regarding the stroke onset, we grouped the observation into five general groups. (1) Observations which compared baseline measures to the first day of the stroke, *baseline vs day one* or acute that covered four observations and 202 participants [32, 39, 59, 60]; (2) Observations comparing the baseline measures to the peripheral level of BDNF within the first week of stroke onset (mostly 3–7 days) *baseline vs week one or sub-acute,* consisting of 14 observations and 1366 patients [32, 39, 42, 44, 54, 56, 59, 61–67]; (3) Observations investigating the difference between baseline levels of BDNF and day 30 or above, *baseline vs. over one month or chronic*, that included 11 observations and 1093 participants [16, 39, 43, 54, 56, 65–69]; (4) Comparison of the first day of the stroke to the first week after the onset, *Day 1 vs Week 1* consisting three observations and 162 participants [32, 39, 59]; (5) Lastly the seven observations investigating the BDNF pattern, in 855 participants, within the first week of stroke and over the one months of affecting the patients [39, 54, 56, 65–67, 70].

Based on the high heterogeneity level detected with I^2^ (over 74%) in every group, we conducted a random effect model analysis separately for each above mentioned observational group. No significant difference were addressed in the SMD in any of the included groups. Nor any publication bias was found (Additional file 2: Fig. S2, Additional file 3: Fig. S3, Additional file 4: Fig. S4, Additional file 5: Fig. S5, Additional file 6: Fig. S6, Additional file 7: Fig. S7, Additional file 8: Fig. S8, Additional file 9: Fig. S9, Additional file 10: Fig. S10, Additional file 11: Fig. S11).

### Effect of depression on serum BDNF levels in PwS

**Fig. 5:**
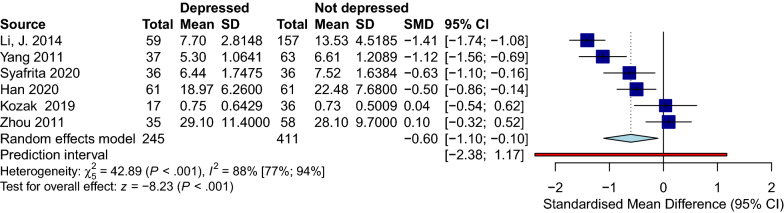
Forest depression

**Fig. 6:**
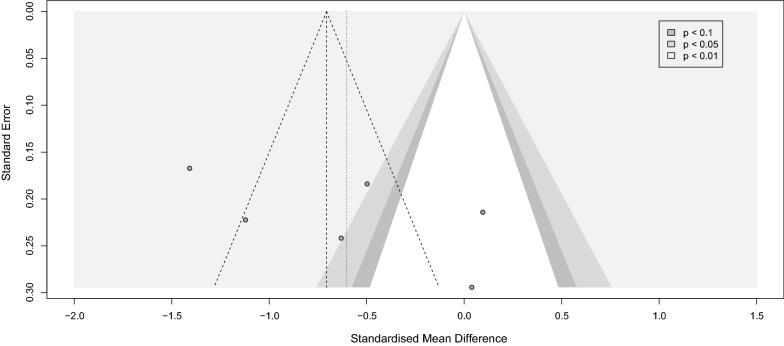
Funnel depression

Six trials with 411 patients and 245 patients without depression compared serum BDNF between these two groups [43, 44, 71–74]. All the studies followed Diagnostic and Statistical Manual of Mental Disorders 4th version (DSM-IV) criteria to assess their sample for depression. Hamilton Depression Rating Scale (HDRS) was also used by four studies [43, 44, 73, 74]. While every included article assessed PSD, only two addressed the history of depression in their patients before the stroke [72, 73]. Patients with depression had significantly lower levels of BDNF than the participant in the non-depressed group (SMD [95%CI] = − 0.60 [− 1.10 to − 0.10], *p-*value < 0.001, I^2^ = 88%, *p-*value < 0.001) (Fig. 5). No publication bias was detected. Due to the low number of included studies, we could not conduct meta-regression or sensitivity analysis (Fig. 6).

### Effect of physical training on serum BDNF levels in PwS

Sixteen studies with 738 patients provided original data on BDNF concentration before and after applying a specific physical training protocol. The training modality included regular physiotherapy sessions [75–77], aerobic exercises [78–80], high-intensity interval training (HIIT) [81], and proprioceptive neuromuscular facilitation exercise (PNF) [82] (Details are illustrated in Table 3). In addition, the number of sessions varied among the included studies starting from only one session [78] to as many as 56 sessions [16]. Also, the duration of sessions ranged from 12 min [83] to 120 min [75]. 13 observational groups were included in the immediate analysis, and eleven observational groups were in the delayed analysis.

#### Sample collection immediately after physical training

**Fig. 7:**
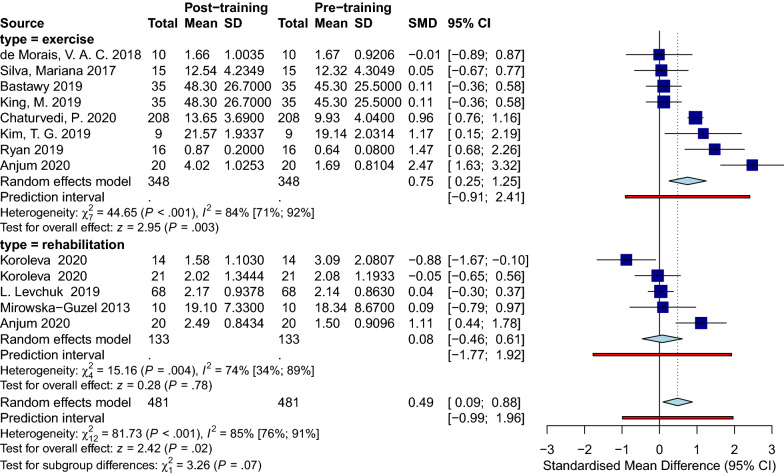
Forest immediate

**Fig. 8:**
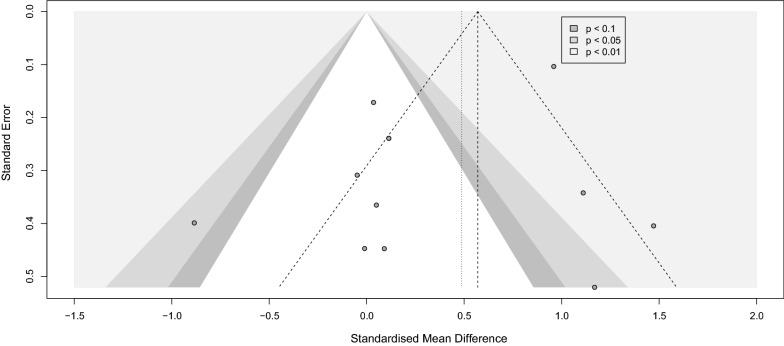
Funnel immediate

Analysis for the immediate group showed a positive effect of physical training in general on BDNF level immediately after the intervention with (SMD [95%CI] = 0.49 [0.09 to 0.88]), *p-value* = 0.02, I^2^ = 85%, *p-value* < 0.001) (Fig. 7). Within the immediate group, we did a subgroup analysis on different training modalities. Eight observations that performed any sort of exercise fell into our exercise subgroup, and five observations applying regular rehabilitation or physiotherapy were categorized as rehabilitation subgroup. The subgroup analysis showed that only in the exercise group BDNF levels significantly increased immediately after physical training (SMD [95%CI] = 0.75 [0.25 to 1.25], *p-value* = 0.003). No subgroup differences were detected. Sensitivity analysis showed that the study of Anjum et al. [84] was influential (Additional file 12: Fig. S12). After omitting this record, the overall effect size did not remain significant (SMD [95%CI] = 0.34 [− 0.03 to 0.71], I^2^ = 82%) (Additional file 13: Fig. S13). Lastly, publication bias was not observed between the included studies (Fig. 8).

#### Sample collection with a delay after physical training

**Fig. 9:**
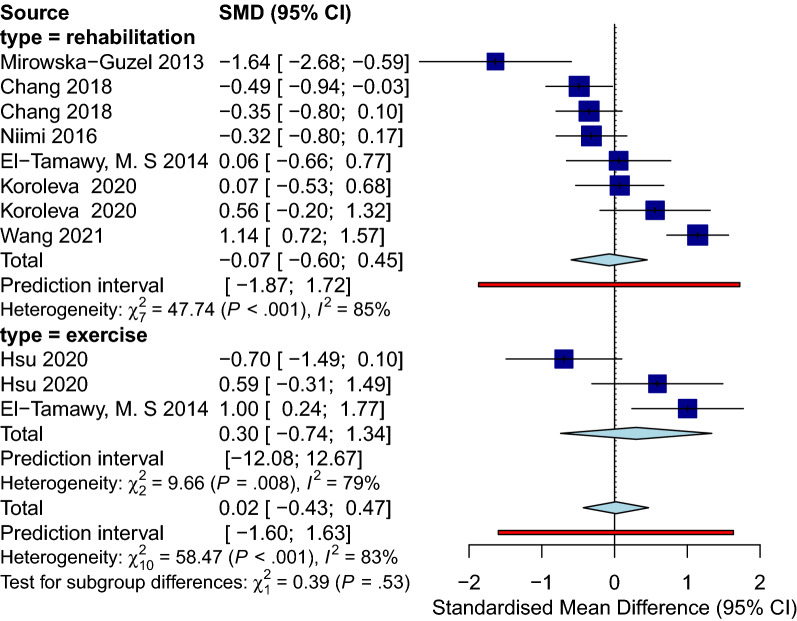
Forest delayed

**Fig. 10:**
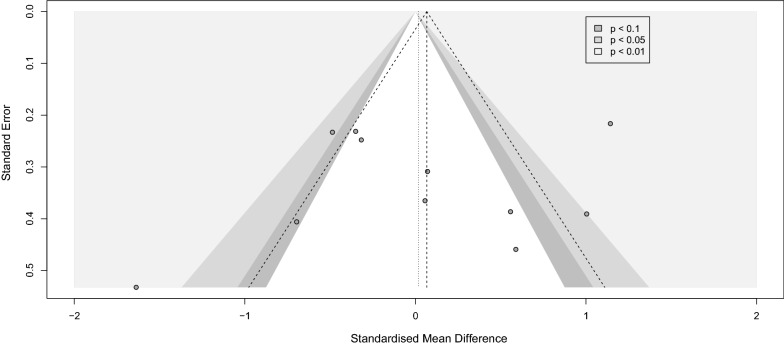
Funnel delayed

The analysis of the delayed group consisting of eleven observations showed no significant effect of the intervention at this delayed phase of sample collection (SMD [95%CI] = 0.02 [− 0.43 to 0.47], I^2^ = 83%) (Fig. 9). Similarly, BDNF levels did not change significantly in any of the subgroups. Leave-one-out analysis showed that after omission of Wang et al., 2021 study [16], the I^2^ index reduced to 68% while the overall effect size remained not significant (Additional file 14: Fig. S14 and Additional file 15: Fig. S15). No publication bias in either group was noted (Fig. 10).

### Repeated transcranial magnetic stimulation (rTMS)

**Fig. 11:**
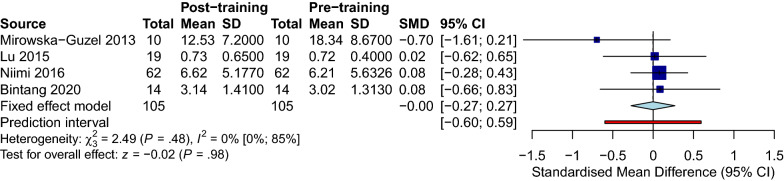
rtms stroke corrected

**Fig. 12:**
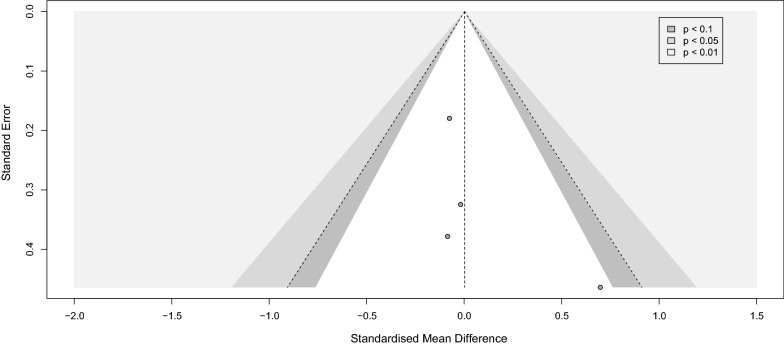
Funnel rtms

Four studies measured the BDNF levels in 105 PwS after receiving rTMS; Lu 2015 used plasma while the other three used serum samples [56, 75, 77, 85]. The overall meta-analysis of the studies revealed no difference in the BDNF levels between the rTMS and the sham stimulation group (SMD [95%CI] = 0.00 [− 0.27 to 0.27]). No significant heterogeneity was found (I^2^ = 0%, *p-value* = 0.48) (Fig. 11). Meta bias in the included studies is presented visually in the funnel plot (Fig. 12), with Begg’s and Egger’s tests of 0.0603 and 0.081, respectively.

## Discussion

With this study, we confirm that the BDNF level is significantly lower in the patients with stroke than in the healthy controls. Interestingly, it is also lower in patients with post-stroke depression compared to PwS without depression. These results are compatible with the hypothesis that a lower level of BDNF is associated with the pathogenesis of neural loss in several neuropsychiatric disorders and cognitive deficits [12, 86–91]. Although the severity and type of stroke, either ischemic or hemorrhagic, can possibly correlate with the BDNF level as addressed by Chaturvedi et al. [82], the lack of reporting data from the included studies prohibited us from further regression analysis of these influential factors.

We assume that neural rehabilitation following a stroke measured with BDNF happens in an insignificant amount in the absence of additional intervention. Individual differences in the neural recovery have made some fluctuations in the level of BDNF days, weeks and months post-stroke, but have led to no significant differences between various time points in this meta-analysis; the overall BDNF pattern was neither increasing nor decreasing through time. This implies that stroke can lead to an irreversible decrease in the level of BDNF or the rate of neural recovery.

In addition, our analysis also confirmed a positive effect of physical training, regardless of performing exercise or routine physiotherapy or rehabilitation, on the BDNF level immediately after the intervention. In contrast, no significant effect was detected when the BDNF samples were collected after a time point from the intervention (delayed). Moreover, the analysis of the effect of rTMS on PwS showed zero effect on the circulating BDNF.

BDNF protein is a member of nerve growth factors, discovered in 1982 [92]; since then, a strong body of evidence has suggested important roles of BDNF in neurogenesis and synaptic plasticity along with suppressive effects on apoptosis in the central and peripheral nervous system. BDNF works through different signaling mechanisms by binding to its high-affinity receptor, known as tropomyosin receptor kinase B (TrkB) [90, 93]. It appears that three main cascades are involved in BDNF signaling pathways; (1) Ras/MAPK/ERK pathway; (2) IRS-1/PI3K/AKT pathway, and (3) PLC/DAG/IP3 pathway. These pathways result in the regulation of the encoding of various proteins, which are associated with different processes such as neurogenesis, synaptic plasticity, and cell survival [90, 94–96]. All of which are essential for synaptogenesis, restoring the ability of recovery and survival after a neural insult such as stroke [97]. Circulating BDNF is detected and measured chiefly by ELISA kits; However, these kits demonstrate a reasonable difference in mean BDNF measures in the same samples; they differ in the number of mature vs pro BDNF detection. Among the existing kits, the Biosensis and Aviscera-Bioscience, measuring total BDNF and mature BDNF, respectively, are the most performant and recommended for clinical studies. Moreover, plasma samples are sensitive to preparation procedures such as room temperature and varying time needed for platelet lysis, making the pre-procedure time-consuming and leading to different reported measures with changing operators. Thus, it is advised to measure serum BDNF concentration minimally affected by extrinsic factors and about 100 folds higher than plasma levels [98].

Many studies have focused on the alternation of the peripheral BDNF level in different situations and reported BDNF changes in neurodegenerative disorders, brain insults, and psychiatric disorders [12, 89–91, 93]. Stroke as the second leading cause of death and morbidity worldwide is investigated remarkably, specifically in regards to post-stroke recovery measures. A notable number of studies have investigated the impact of numerous modalities such as oral or intravenous medications, physical interventions, and brain stimulation on the BDNF level as an indicator of plasticity in PwS. Within medications, rtPA, antioxidants such as Saffron, statins, and Cytoflavin, have shown promising effects on increasing the BDNF level in each individual study [39, 42, 52, 61], which did not meet the meta-analysis threshold in the current research article (total number of 3 studies). Additionally, specific brain stimulation using rTMS which was assessed by 4 independent studies showed zero effect on BDNF in our meta-analysis.

### Post stroke depression (PSD)

The current study reveals decreased levels of BDNF among the PSD group compared to PwS with no clinical depression. This implies that a decreased BDNF level can be an early predictor of depression in PwS. Since the BDNF is also decreased generally in PwS, there should exist a definite criteria, either a threshold or a difference amount of drop in BDNF level, on which we consider a PwS susceptible to develop PSD. Suggested amount in the current literature are very wide and inconsistent for this matter. Yang et al. [44] mentioned a 28 fold higher risk of depression in PwS with BDNF levels lower than 5.86 ng/ml, while Li et al. [99] report an 11.5-fold increase in the risk of post-stroke depression in case of BDNF < 10.2 ng/ml. An increasing response to BDNF to antidepressant consumption in PwS diagnosed with PSD also suggests the effect of BDNF among this population [100, 101]. Altogether may open the door to the proposal of new therapeutic options in PwS.

### Physical training

We confirm that exercises and rehabilitation programs promote BDNF production and motor recovery following stroke [102, 103]. Literature shows the impacts of training on post-stroke circulating BDNF levels vary based on the program’s duration and intensity [102, 104]. Programs with higher intensity resulted in higher BDNF levels as confirmed by de Morais et al., a significant increase in serum BDNF levels were detected in their moderate exercise group (64–76% of maximum heart rate), not their mild group (50–63% of maximum heart rate) [105]. Moreover, gradually increasing intensity results in a significantly higher increase in serum BDNF [102]. Similar to the exercise, the degree of the rehabilitation program correlated significantly with the BDNF level and motor recovery, supposing a critical threshold in the intensity that the impact could not be detected below that level [106]. As defined in mentioned studies, a threshold exists for the severity of the training that zero benefit is noticed below the point. This makes us assume that the duration of each session would have the same pattern of effect, which is awaiting to be discovered.

Although the duration of the training can potentially alter BDNF levels, none of our included studies has performed a specific, clear comparison for this matter (e.g., comparing the numbers of sessions or the session duration). In regards to comparing different exercise protocols with rehabilitation modalities, El-tamawy et al. suggest that implementing physiotherapy in combination with aerobic exercise leads to higher BDNF levels compared to the routine physiotherapy group [76]. Lastly, the effect of time points in data collection was of remarkable significance. Our analysis confirms a promising short-term effect of exercise on motor recovery by higher levels of circulatory BDNF when assessed immediately after the session, while low to zero long-term impact is detected on BDNF level following exercise. The problem is that the current protocols of treatment are failing to have a sustained effect and the termination of the intervention leads to a decrease in overall benefit. At this point, we need to come up with more effective treatment protocols for the physical training, and this does not imply that the exercise per se is ineffective; rather our existing modality is.

## Conclusion

In conclusion, stroke significantly affects the level of BDNF, which is positively correlated with neural plasticity and post-stroke recovery in various domains such as cognition, affect, and motor function. Our study also confirms the significantly lower levels of BDNF in patients with stroke than the healthy controls, patients with the clinical diagnosis of depression to non-depressed patients and significantly higher levels of BDNF in patients who do moderate to severe physical training. Furthermore, literature imposes different modalities to overcome the effect of BDNF reduction, from which our study confirms the short-term effect of moderate to intense exercise or rehabilitation. We believe that BDNF could be regarded as a valuable diagnostic biomarker for acute stroke and a potential screening factor to observe the effectiveness of treatment. Existing modalities lack a definite protocol by which a maximum benefit is reached. Thus, further studies should focus on addressing the exact dosage of proposed drugs such as antioxidants, antidepressants, statins and a definite intensity and duration for the physical training. Lastly, investigating the role of BDNF supplementation for severe stroke patients could be of great value.

## Supplementary Information

**Additional file1**: **Figure 1**. Influence analysis plot of BDNF levels among PwS vs Healthy controls. The study of Algin et al. 2019 was found influential.

**Additional file 2**: **Figure 2**. Meta-analysis of the BDNF levels in PwS, Baseline vs Day 1. We found no significant difference between the two groups.

**Additional file 3**: **Figure 3**. Meta-analysis of the BDNF levels in PwS, Day 1 vs Week 1. We found no significant difference between the two groups.

**Additional file 4**: **Figure 4**. Meta-analysis of the BDNF levels in PwS, Baseline vs Over 1 month. We found no significant difference between the two groups.

**Additional file 5**: **Figure 5**. Meta-analysis of the BDNF levels in PwS, Baseline vs Week 1. We found no significant difference between the two groups.

**Additional file 6**: **Figure 6**. Meta-analysis of the BDNF levels in PwS, Week 1 vs Over 1 month. We found no significant difference between the two groups.

**Additional file 7**: **Figure 7**. Funnel plot of the studies included in Baseline vs Day 1.

**Additional file 8**: **Figure 8**. Funnel plot of the studies included in Day 1 vs Week 1.

**Additional file 9**: **Figure 9**. Funnel plot of the studies included in Baseline vs Over 1 month.

**Additional file 10**: **Figure 10**. Funnel plot of the studies included in Baseline vs Week 1.

**Additional file 11**: **Figure 11**. Funnel plot of the studies included in Week 1 vs Over 1 month.

**Additional file 12**: **Figure 12**. Influence analysis plot of BDNF levels in physical training subgroup baseline vs. immediate after the training. The study of Anjum et al. 2020 was found influential.

**Additional file 13**: **Figure 13**. Influence analysis, ‘leave one out’ plot, of BDNF levels in physical training subgroup baseline vs. immediate after the training. Omitting the study of Anjum et al. 2020 resulted in a non-significant difference between the two groups.

**Additional file 14**: **Figure 14**. Influence analysis plot of BDNF levels in physical training subgroup baseline vs. with a delayed period after the training. No influential study was found.

**Additional file 15**: **Figure 15**. Influence analysis, ‘leave one out’ plot, of BDNF levels in physical training subgroup baseline vs. with a delayed period after the training. After omitting the study of Wang et al. 2021 the I^2^ index reduced to 68%.

## Data Availability

The excel sheet for the extracted variables is available upon request.

## Abbreviations

DALY: Disability-adjusted life years
PwS: Patients with stroke
BDNF: Brain-derived neurotrophic factor
SD: Standard deviation
SMD: Standardized mean difference
CI: Confidence interval
rTMS: Repeated transcranial magnetic stimulation
PT: Physical training
HIIT: High-intensity interval training
PNF: Proprioceptive neuromuscular facilitation exercise
PSD: Post-stroke depression

## References

[1] M Patrice Lindsay (Corresponding Author)1 BN, Ralph L. Sacco3, Michael Brainin4, Werner Hacke5, Sheila Martins6, Jeyaraj Pandian7, Valery Feigin. Global Stroke Fact Sheet 2019. World Stroke Organization (WSO). 2020:https://www.world-stroke.org/assets/downloads/WSO_Fact-sheet_15.01.2020.pdf. 10.1177/1747493019881353 31658892

[2] Feigin VL, Nichols E, Alam T, Bannick MS, Beghi E, Blake N (2019). Global, regional, and national burden of neurological disorders, 1990–2016: a systematic analysis for the Global Burden of Disease Study 2016. Lancet Neurol.

[3] Pikula A, Beiser AS, Chen TC, Preis SR, Vorgias D, DeCarli C (2013). Serum brain-derived neurotrophic factor and vascular endothelial growth factor levels are associated with risk of stroke and vascular brain injury framingham study. Stroke.

[4] Li WY, Ling SC, Yang Y, Hu ZY, Davies H, Fang MR (2014). Systematic hypothesis for post-stroke depression caused inflammation and neurotransmission and resultant on possible treatments. Neuroendocrinol Lett.

[5] Ploughman M, Eskes GA, Kelly LP, Kirkland MC, Devasahayam AJ, Wallack EM (2019). Synergistic benefits of combined aerobic and cognitive training on fluid intelligence and the role of IGF-1 in chronic stroke. Neurorehabil Neural Repair.

[6] Huang EJ, Reichardt LF (2001). Neurotrophins: roles in neuronal development and function. Annu Rev Neurosci.

[7] Schinder AF, Poo M-M (2000). The neurotrophin hypothesis for synaptic plasticity. Trends Neurosci.

[8] Tadi P, Lui F. Acute Stroke. StatPearls. Treasure Island (FL): StatPearls Publishing Copyright © 2021, StatPearls Publishing LLC.; 2021.

[9] Diniz BS, Teixeira AL (2011). Brain-derived neurotrophic factor and Alzheimer’s disease: physiopathology and beyond. NeuroMol Med.

[10] Ferreira RN, de Miranda AS, Rocha NP, Simoes e Silva AC, Teixeira AL, da Silva Camargos ER (2018). Neurotrophic factors in Parkinson's disease: what have we learned from pre-clinical and clinical studies?. Curr Med Chem.

[11] Halepoto DM, Bashir S, Al-Ayadhi L (2014). Possible role of brain-derived neurotrophic factor (BDNF) in autism spectrum disorder: current status. J Coll Phys Surg Pak..

[12] Mojtabavi H, Saghazadeh A, van den Heuvel L, Bucker J, Rezaei N (2020). Peripheral blood levels of brain-derived neurotrophic factor in patients with post-traumatic stress disorder (PTSD): A systematic review and meta-analysis. PLoS ONE.

[13] Hassan TM, Yarube IU (2018). Peripheral brain-derived neurotrophic factor is reduced in stroke survivors with cognitive impairment. Pathophysiology.

[14] Luo W, Liu T, Li S, Wen H, Zhou F, Zafonte R (2019). The serum BDNF level offers minimum predictive value for motor function recovery after stroke. Transl Stroke Res.

[15] Stanne TM, Åberg ND, Nilsson S, Jood K, Blomstrand C, Andreasson U (2016). Low circulating acute brain-derived neurotrophic factor levels are associated with poor long-term functional outcome after ischemic stroke. Stroke.

[16] Wang H, Zhu C, Liu D, Wang Y, Zhang J, Wang S (2021). Rehabilitation training improves cognitive disorder after cerebrovascular accident by improving BDNF Bcl-2 and Bax expressions in regulating the JMK pathway. Eur Rev Med Pharmacol Sci.

[17] Graph reader. http://www.graphreader.com/.

[18] Whiting PF, Rutjes AW, Westwood ME, Mallett S, Deeks JJ, Reitsma JB (2011). QUADAS-2: a revised tool for the quality assessment of diagnostic accuracy studies. Ann Intern Med.

[19] Huedo-Medina TB, Sanchez-Meca J, Marin-Martinez F, Botella J (2006). Assessing heterogeneity in meta-analysis: Q statistic or I2 index?. Psychol Methods.

[20] DerSimonian R, Laird N (1986). Meta-analysis in clinical trials. Control Clin Trials.

[21] Borenstein M, Hedges LV, Higgins JP, Rothstein HR (2010). A basic introduction to fixed-effect and random-effects models for meta-analysis. Res Synth Methods.

[22] Li B, Piao CS, Liu XY, Guo WP, Xue YQ, Duan WM (2010). Brain self-protection: The role of endogenous neural progenitor cells in adult brain after cerebral cortical ischemia. Brain Res.

[23] Reynolds MA, Kirchick HJ, Dahlen JR, Anderberg JM, McPherson PH, Nakamura KK (2003). Early biomarkers of stroke. Clin Chem.

[24] Egger M, Davey Smith G, Schneider M, Minder C (1997). Bias in meta-analysis detected by a simple, graphical test. BMJ.

[25] Begg CB, Mazumdar M (1994). Operating characteristics of a rank correlation test for publication bias. Biometrics.

[26] Kostenko EV (2017). Influence chronopharmacology therapy methionine (Melaxen) on the dynamics of sleep disturbance, cognitive and emotional disorders, brain-derived neurotrophic factor (BDNF) in patients with cerebral stroke in the early and late recovery periods. Zhurnal Nevrologii i Psihiatrii imeni SS Korsakova.

[27] Tokami H, Ago T, Sugimori H, Kuroda J, Awano H, Suzuki K (2013). RANTES has a potential to play a neuroprotective role in an autocrine/paracrine manner after ischemic stroke. Brain Res.

[28] Boyne P, Meyrose C, Westover J, Whitesel D, Hatter K, Reisman DS (2019). Exercise intensity affects acute neurotrophic and neurophysiological responses poststroke. J Appl Physiol.

[29] Cichon N, Bijak M, Czarny P, Miller E, Synowiec E, Sliwinski T (2018). Increase in blood levels of growth factors involved in the neuroplasticity process by using an extremely low frequency electromagnetic field in post-stroke patients. Front Aging Neurosci..

[30] Casas S, Perez AF, Mattiazzi M, Lopez J, Folgueira A, Gargiulo-Monachelli GM (2017). Potential biomarkers with plasma cortisol, brain-derived neurotrophic factor and nitrites in patients with acute ischemic stroke. Curr Neurovasc Res.

[31] Jiménez I, Sobrino T, Brea D, Cristobo I, Rodríguez-Yáñez M, Blanco M (2008). Molecular markers of inflammation in post-stroke depression. Trauma.

[32] Di Lazzaro V, Profice R, Pilato F, Dileone M, Florio L, Tonali PA (2007). BDNF plasma levels in acute stroke. Neurosci Lett.

[33] Greisenegger S, Segal HC, Burgess AI, Poole DL, Mehta Z, Rothwell PM (2015). Biomarkers and mortality after transient ischemic attack and minor ischemic stroke population-based study. Stroke.

[34] Hidayat A, Arief M, Wijaya A, As'ad S (2016). Vascular endothelial growth factor and brain-derived neurotrophic factor levels in ischemic stroke subject. Indonesian Biomed J.

[35] Kotlega D, Zembron-Lacny A, Morawin B, Golab-Janowska M, Nowacki P, Szczuko M (2020). Free fatty acids and their inflammatory derivatives affect BDNF in stroke patients. Mediat Inflam.

[36] Kozak HH, Uguz F, Kilinc I, Uca AU, Tokgoz OS, Akpinar Z (2017). Delirium in patients with acute ischemic stroke admitted to the non-intensive stroke unit: Incidence and association between clinical features and inflammatory markers. Neurol Neurochir Pol.

[37] Lasek-Bal A, Jedrzejowska-Szypulka H, Rozycka J, Bal W, Holecki M, Dulawa J (2015). Low Concentration of BDNF in the acute phase of ischemic stroke as a factor in poor prognosis in terms of functional status of patients. Med Sci Monit.

[38] Lasek-Bal A, Jedrzejowska-Szypulka H, Student S, Warsz-Wianecka A, Zareba K, Puz P (2019). The importance of selected markers of inflammation and blood-brain barrier damage for short-term ischemic stroke prognosis. J Physiol Pharmacol.

[39] Rodier M, Quirie A, Prigent-Tessier A, Bejot Y, Jacquin A, Mossiat C (2015). Relevance of Post-Stroke Circulating BDNF Levels as a Prognostic Biomarker of Stroke Outcome. Impact of rt-PA Treatment. PLoS ONE.

[40] Siotto M, Aprile I, Simonelli I, Pazzaglia C, Ventriglia M, Santoro M (2017). An exploratory study of BDNF and oxidative stress marker alterations in subacute and chronic stroke patients affected by neuropathic pain. J Neural Transm.

[41] Widodo J, Asadul A, Wijaya A, Lawrence G (2016). Correlation between Nerve Growth Factor (NGF) with Brain Derived Neurotropic Factor (BDNF) in Ischemic Stroke Patient. Bali Medical Journal.

[42] Zhang JM, Mu XL, Breker DA, Li Y, Gao ZL, Huang YL (2017). Atorvastatin treatment is associated with increased BDNF level and improved functional recovery after atherothrombotic stroke. Int J Neurosci.

[43] Zhou Z, Lu T, Xu G, Yue X, Zhu W, Ma M (2011). Decreased serum brain-derived neurotrophic factor (BDNF) is associated with post-stroke depression but not with BDNF gene Val66Met polymorphism. Clin Chem Lab Med.

[44] Yang L, Zhang Z, Sun D, Xu Z, Yuan Y, Zhang X (2011). Low serum BDNF may indicate the development of PSD in patients with acute ischemic stroke. Int J Geriatr Psychiatry.

[45] Wang J, Huang Q, Ding J, Wang X (2019). Elevated serum levels of brain-derived neurotrophic factor and miR-124 in acute ischemic stroke patients and the molecular mechanism. Biotech.

[46] Wang J, Gao L, Yang YL, Li YQ, Chang T, Man MH (2017). Low serum levels of brain-derived neurotrophic factor were associated with poor short-term functional outcome and mortality in acute ischemic stroke. Mol Neurobiol.

[47] Stanne TM, Aberg ND, Nilsson S, Jood K, Blomstrand C, Andreasson U (2016). Low circulating acute brain-derived neurotrophic factor levels are associated with poor long-term functional outcome after ischemic stroke. Stroke.

[48] Santos GL, Alcântara CC, Silva-Couto MA, García-Salazar LF, Russo TL (2016). Decreased brain-derived neurotrophic factor serum concentrations in chronic post-stroke subjects. J Stroke Cerebrovasc Dis.

[49] Qiao H-J, Li Z-Z, Wang L-M, Sun W, Yu J-C, Wang B (2017). Association of lower serum Brain-derived neurotrophic factor levels with larger infarct volumes in acute ischemic stroke. J Neuroimmunol.

[50] Pascotini ET, Flores AE, Kegler A, Konzen V, Fornari AL, Arend J (2018). Brain-derived neurotrophic factor levels are lower in chronic stroke patients: a relation with manganese-dependent superoxide dismutase ALA16VAL single nucleotide polymorphism through tumor necrosis factor-alpha and caspases pathways. J Stroke Cerebrovasc Dis.

[51] Koroleva E, Alifirova V, Latypova A, Ivanova S, Levchuk L, Kazakov S (2019). Correlation between neurological deficit and serum BDNF in patients with ischemic stroke after early rehabilitation. Eur J Neurol.

[52] Karakulova YV, Selyanina NV, Zhelnin AV, Filimonova TA, Tsepilov SV (2018). Effects of antioxidant treatment on neurotrophins and rehabilitation processes following stroke. Neurosci Behav Physiol.

[53] Huțanu A, Iancu M, Maier S, Bălaşa R, Dobreanu M (2020). Plasma biomarkers as potential predictors of functional dependence in daily life activities after ischemic stroke: a single center study. Ann Indian Acad Neurol.

[54] Chan A, Yan J, Csurhes P, Greer J, McCombe P (2015). Circulating brain derived neurotrophic factor (BDNF) and frequency of BDNF positive T cells in peripheral blood in human ischemic stroke: effect on outcome. J Neuroimmunol.

[55] Algin A, Erdogan MO, Aydin I, Poyraz MK, Sirik M (2019). Clinical usefulness of brain-derived neurotrophic factor and visinin-like protein-1 in early diagnostic tests for acute stroke. Am J Emerg Med.

[56] Lu HT, Zhang T, Wen M, Sun L (2015). Impact of repetitive transcranial magnetic stimulation on post-stroke dysmnesia and the role of BDNF Val66Met SNP. Med Sci Monit.

[57] Chen HG, Wang M, Jiao AH, Tang G, Zhu W, Zou P (2018). Research on changes in cognitive function, beta-amyloid peptide and neurotrophic factor in stroke patients. Eur Rev Med Pharmacol Sci.

[58] Algin A, Erdogan MO, Aydin I, Poyraz MK, Sirik M (2019). Clinical usefulness of brain-derived neurotrophic factor and visinin-like protein-1 in early diagnostic tests for acute stroke. Am J Emerg Med.

[59] Hutanu A, Iancu M, Maier S, Balasa R, Dobreanu M (2020). Plasma biomarkers as potential predictors of functional dependence in daily life activities after ischemic stroke: a single center study. Ann Indian Acad Neurol.

[60] Pedarci M, Breniere C, Perne N, Vergely C, Bejot Y, Marie C (2018). Brain-derived neurotrophic factor in peripheral blood mononuclear cells and stroke outcome. Exp Biol Med.

[61] Asadollahi M, Nikdokht P, Hatef B, Sadr SS, Sahraei H, Assarzadegan F (2019). Protective properties of the aqueous extract of saffron (*Crocus sativus* L.) in ischemic stroke, randomized clinical trial. J Ethnopharmacol.

[62] Mourao AM, Vicente LCC, Abreu MNS, Sant'Anna RV, Vieira ELM, de Souza LC (2019). Plasma levels of brain-derived neurotrophic factor are associated with prognosis in the acute phase of ischemic stroke. J Stroke Cerebrovasc Dis.

[63] Otero-Ortega L, Gutierrez-Fernandez M, Gutierrez-Zuniga R, Madero-Jarabo R, de Lecinana MA, Laso-Garcia F (2019). The effect of post-stroke hyperglycaemia on the levels of brain damage and repair-related circulating biomarkers: the Glycaemia in Acute Stroke Study II. Eur J Neurol.

[64] Billinger SA, Sisante JFV, Whitaker AA, Abraham MG (2018). Time course of flow-mediated dilation and vascular endothelial growth factor following acute stroke. J Stroke Cerebrovasc Dis.

[65] Lopez-Cancio E, Ricciardi AC, Sobrino T, Cortes J, de la Ossa NP, Millan M (2017). Reported prestroke physical activity is associated with vascular endothelial growth factor expression and good outcomes after stroke. J Stroke Cerebrovasc Dis.

[66] Roslavtceva V, Bushmelev E, Astanin P, Zabrodskaya T, Salmina A, Prokopenko S, et al. Blood plasma trophic growth factors predict the outcome in patients with acute ischemic stroke. 2020. p. 27–39.

[67] Sobrino T, Rodriguez-Yanez M, Campos F, Iglesias-Rey R, Millan M, de la Ossa NP (2020). Association of high serum levels of growth factors with good outcome in ischemic stroke: a multicenter study. Transl Stroke Res.

[68] Bembenek JP, Kurczych K, Klysz B, Cudna A, Antczak J, Czlonkowska A (2020). Prediction of recovery and outcome using motor evoked potentials and brain derived neurotrophic factor in subacute stroke. J Stroke Cerebrovasc Dis.

[69] Bintang AK, Akbar M, Amran MY, Hammado N (2020). The effect of high-and low-frequency repetitive transcranial magnetic stimulation therapy on serum brain-derived neurotropic factor level and motor ability in ischemic stroke patients: A single-center study. Open Access Macedonian J Med Sci..

[70] Prodjohardjono A, Sutarni S, Setyopranoto I (2020). Serum Brain-Derived Neurotrophic Factor (BDNF) Level May Predict the Functional Outcome of Acute Ischemic Stroke Patients. Biomed Pharmacol J..

[71] Han ZX, Wang Y, Qi LL, Wang JN, Wong O, Chen JH (2020). Differential Association of Serum BDNF with poststroke depression and poststroke anxiety. Arch Phys Med Rehabil.

[72] Kozak HH, Uguz F, Kilinc I, Uca AU, Tokgoz OS, Guney F (2019). A cross-sectional study to assess the association between major depression and inflammatory markers in patients with acute ischemic stroke. Indian J Psychiatry.

[73] Li J, Zhao YD, Zeng JW, Chen XY, Wang RD, Cheng SY (2014). Serum Brain-derived neurotrophic factor levels in post-stroke depression. J Affect Disord.

[74] Syafrita Y, Amir D, Susanti R, Fadhilah I (2020). Relationship of brain-derived neurotrophic factor, malondialdehyde, and 8-hydroxy 2-deoxyguanosine with post-ischemic stroke depression. Dementia e Neuropsychologia.

[75] Chang WH, Shin MA, Lee A, Kim H, Kim YH (2018). Relationship between Serum BDNF Levels and Depressive Mood in Subacute Stroke Patients: A Preliminary Study. Int J Mol Sci.

[76] El-Tamawy MS, Abd-Allah F, Ahmed SM, Darwish MH, Khalifa HA (2014). Aerobic exercises enhance cognitive functions and brain derived neurotrophic factor in ischemic stroke patients. NeuroRehabilitation.

[77] Mirowska-Guzel D, Gromadzka G, Seniow J, Lesniak M, Bilik M, Waldowski K (2013). Association between BDNF-196 G > A and BDNF-270 C > T polymorphisms, BDNF concentration, and rTMS-supported long-term rehabilitation outcome after ischemic stroke. NeuroRehabilitation.

[78] Kim TG, Bae SH, Kim KY (2019). Effects of dual-task training with different intensity of aerobic exercise on cognitive function and neurotrophic factors in chronic stroke patients. Res J Pharm Technol.

[79] Ryan AS, Xu HC, Ivey FM, Macko RF, Hafer-Macko CE (2019). Brain-derived neurotrophic factor, epigenetics in stroke skeletal muscle, and exercise training. Neurol-Genetics..

[80] Silva M, Morais V, Santos R, Rocha N, Christo P, Fuscaldi L (2017). No change in brain-derived neurotrophic factor levels following a single session of light to-moderate intensity walk in chronic stroke patients. J Neurol Dis..

[81] Hsu CC, Fu TC, Huang SC, Chen CPC, Wang JS (2020). Increased serum brain-derived neurotrophic factor with high-intensity interval training in stroke patients: A randomized controlled trial. Ann Phys Rehab Med..

[82] Chaturvedi P, Singh AK, Tiwari V, Thacker AK (2020). Post-stroke BDNF concentration changes following proprioceptive neuromuscular facilitation (PNF) exercises. J Fam Med Primary Care.

[83] Bastawy S, Ahmed AM (2019). The role of serum levels of insulin-like growth factor-1 and brain-derived neurotrophic factor in predicting recovery in stroke. Egyp J Hosp Med..

[84] Anjum AF, Khan HF, Sadiq N, Samad A, Jawwad G, Ayaz H (2020). Effects of virtual rehabilitation and constraint induced movement therapy on brain derived neurotrophic factorm ediated motor improvement in stroke patients. J Bahria Univ Med Dent Coll.

[85] Niimi M, Hashimoto K, Kakuda W, Miyano S, Momosaki R, Ishima T (2016). Role of brain-derived neurotrophic factor in beneficial effects of repetitive transcranial magnetic stimulation for upper limb hemiparesis after stroke. PLoS ONE.

[86] Dell'Osso L, Carmassi C, Del Debbio A, Dell'Osso MC, Bianchi C, Da Pozzo E (2009). Brain-derived neurotrophic factor plasma levels in patients suffering from post-traumatic stress disorder. Prog Neuropsychopharmacol Biol Psychiatry.

[87] Giacobbo BL, Doorduin J, Klein HC, Dierckx RA, Bromberg E, de Vries EF (2019). Brain-derived neurotrophic factor in brain disorders: focus on neuroinflammation. Mol Neurobiol.

[88] Rahmani F, Saghazadeh A, Rahmani M, Teixeira AL, Rezaei N, Aghamollaii V (2019). Plasma levels of brain-derived neurotrophic factor in patients with Parkinson disease: a systematic review and meta-analysis. Brain Res.

[89] Saghazadeh A, Rezaei N (2017). Brain-derived neurotrophic factor levels in autism: a systematic review and meta-analysis. J Autism Develop Disord.

[90] Bathina S, Das UN (2015). Brain-derived neurotrophic factor and its clinical implications. Arch Med Sci.

[91] Karantali E, Kazis D, Papavasileiou V, Prevezianou A, Chatzikonstantinou S, Petridis F (2021). Serum BDNF levels in acute stroke: a systematic review and meta-analysis. Medicina.

[92] Barde Y-A, Edgar D, Thoenen H (1982). Purification of a new neurotrophic factor from mammalian brain. EMBO J.

[93] Binder DK, Scharfman HE (2004). Brain-derived neurotrophic factor. Growth Factors (Chur, Switzerland).

[94] Dimitropoulou A, Bixby JL (2000). Regulation of retinal neurite growth by alterations in MAPK/ERK kinase (MEK) activity. Brain Res.

[95] Pezet S, Malcangio M, McMahon SB (2002). BDNF: a neuromodulator in nociceptive pathways?. Brain Res Rev.

[96] Vaillant A, Mazzoni I, Tudan C, Boudreau M, Kaplan D, Miller F (1999). Depolarization and neurotrophins converge on the phosphatidylinositol 3-kinase–Akt pathway to synergistically regulate neuronal survival. J Cell Biol.

[97] Wlodarczyk L, Szelenberger R, Cichon N, Saluk-Bijak J, Bijak M, Miller E (2021). Biomarkers of angiogenesis and neuroplasticity as promising clinical tools for stroke recovery evaluation. Int J Mol Sci.

[98] Polacchini A, Metelli G, Francavilla R, Baj G, Florean M, Mascaretti LG (2015). A method for reproducible measurements of serum BDNF: comparison of the performance of six commercial assays. Sci Rep.

[99] Li J, Zhao Y-D, Zeng J-W, Chen X-Y, Wang R-D (2014). Serum Brain-derived neurotrophic factor levels in post-stroke depression. Stroke.

[100] Sen S, Duman R, Sanacora G (2008). Serum brain-derived neurotrophic factor, depression, and antidepressant medications: meta-analyses and implications. Biol Psychiatry.

[101] Shimizu E, Hashimoto K, Okamura N, Koike K, Komatsu N, Kumakiri C (2003). Alterations of serum levels of brain-derived neurotrophic factor (BDNF) in depressed patients with or without antidepressants. Biol Psychiatry.

[102] Sun J, Ke Z, Yip SP, Hu XL, Zheng XX, Tong KY (2014). Gradually increased training intensity benefits rehabilitation outcome after stroke by BDNF upregulation and stress suppression. Biomed Res Int.

[103] Ploughman M, Windle V, MacLellan CL, White N, Dore JJ, Corbett D (2009). Brain-derived neurotrophic factor contributes to recovery of skilled reaching after focal ischemia in rats. Stroke.

[104] Alcantara CC, Garcia-Salazar LF, Silva-Couto MA, Santos GL, Reisman DS, Russo TL (2018). Post-stroke BDNF concentration changes following physical exercise: a systematic review. Front Neurol.

[105] de Morais VAC, Tourino MFD, Almeida ACD, Albuquerque TBD, Linhares RC, Christo PP (2018). A single session of moderate intensity walking increases brain-derived neurotrophic factor (BDNF) in the chronic post-stroke patients. Top Stroke Rehabil.

[106] MacLellan CL, Keough MB, Granter-Button S, Chernenko GA, Butt S, Corbett D (2011). A critical threshold of rehabilitation involving brain-derived neurotrophic factor is required for poststroke recovery. Neurorehabil Neural Repair.

