# Supplementary figures and images for: Circulating brain-derived neurotrophic factor as a potential biomarker in stroke: a systematic review and meta-analysis
Source: J Transl Med. 2022 Mar 14;20:126. doi: 10.1186/s12967-022-03312-y (PMC8919648; doi:10.1186/s12967-022-03312-y)

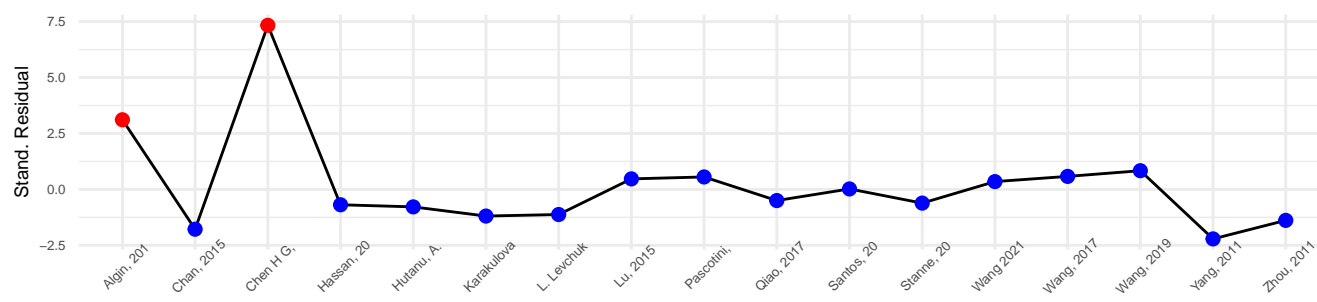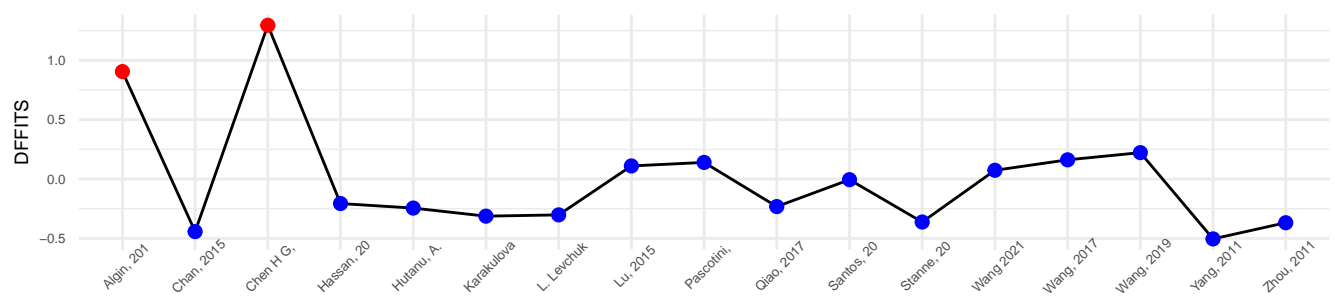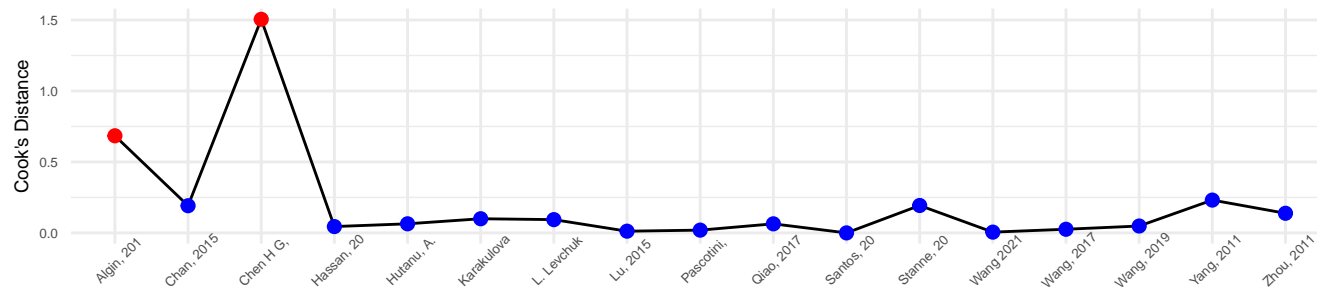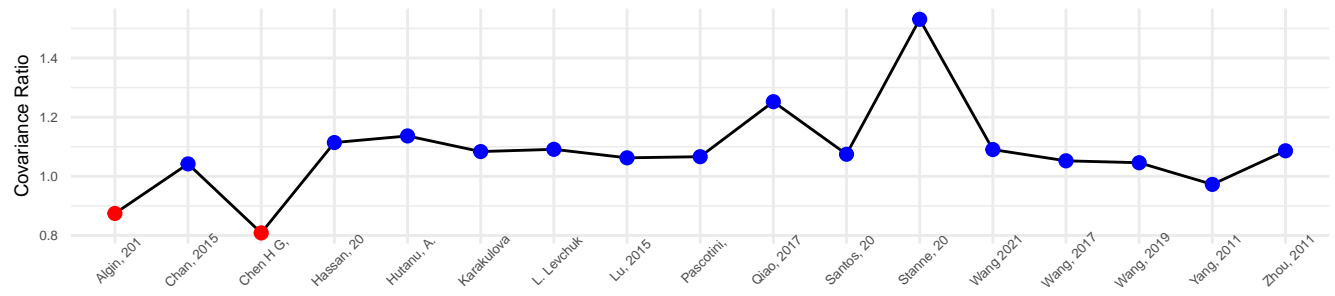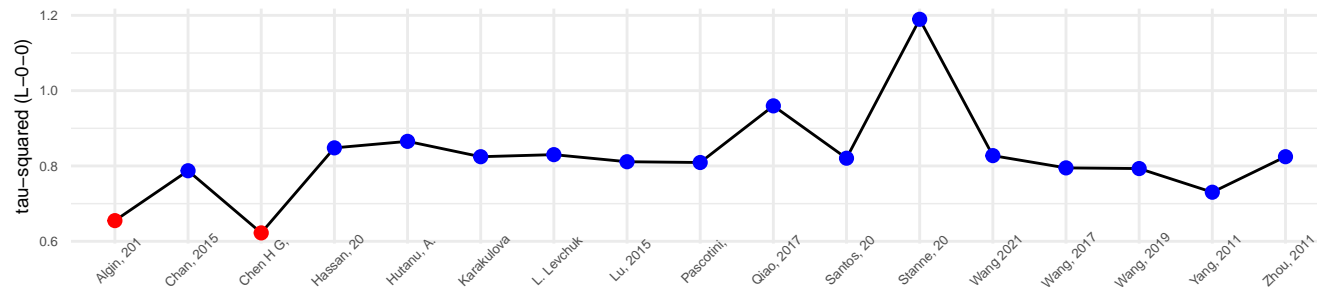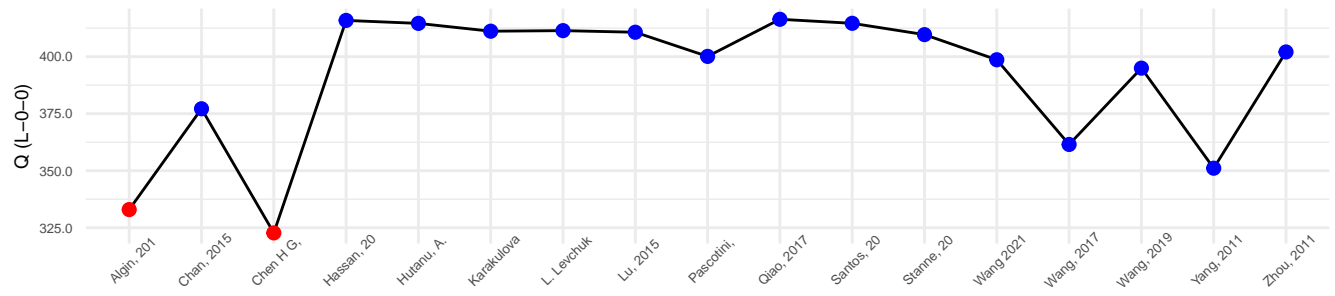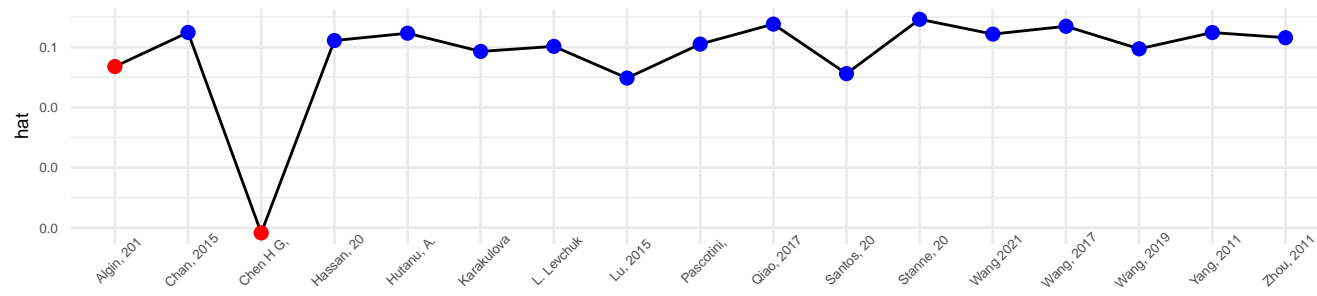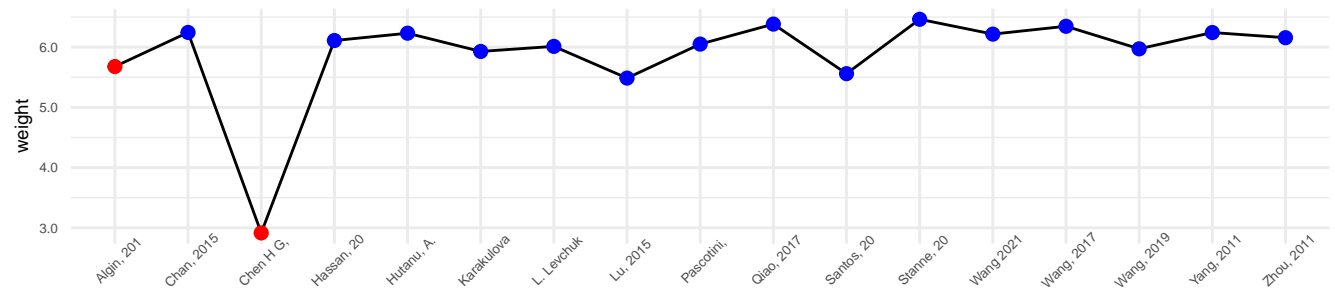

Supplement: Supplementary file 1 — Additional file1: Figure 1. Influence analysis plot of BDNF levels among PwS vs Healthy controls. The study of Algin et al. 2019 was found influential. [file 12967_2022_3312_MOESM1_ESM.pdf]

| Source                                                         | SMD (95% CI)          |
|----------------------------------------------------------------|-----------------------|
| Rodier, M. 2015                                                | -1.11 [ -1.60; -0.63] |
| Di Lazzaro 2007                                                | -0.09 [ -0.97; 0.79]  |
| Hutanu, A. 2020                                                | 0.26 [ 0.00; 0.52]    |
| Total                                                          | -0.31 [ -1.29; 0.67]  |
| Prediction interval                                            | [-12.44; 11.81]       |
| Heterogeneity: $\chi^2_2 = 23.89$ ( $P < .001$ ), $I^2 = 92\%$ |                       |

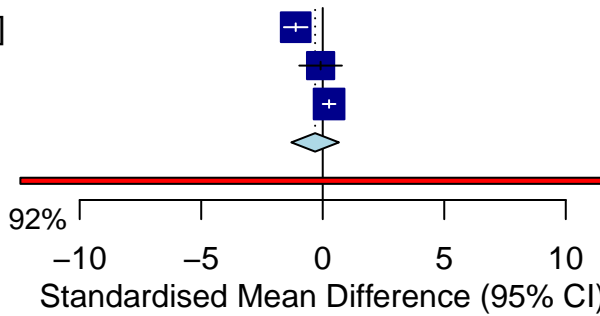

Supplement: Supplementary file 3 — Additional file 3: Figure 3. Meta-analysis of the BDNF levels in PwS, Day 1 vs Week 1. We found no significant difference between the two groups. [file 12967_2022_3312_MOESM3_ESM.pdf]

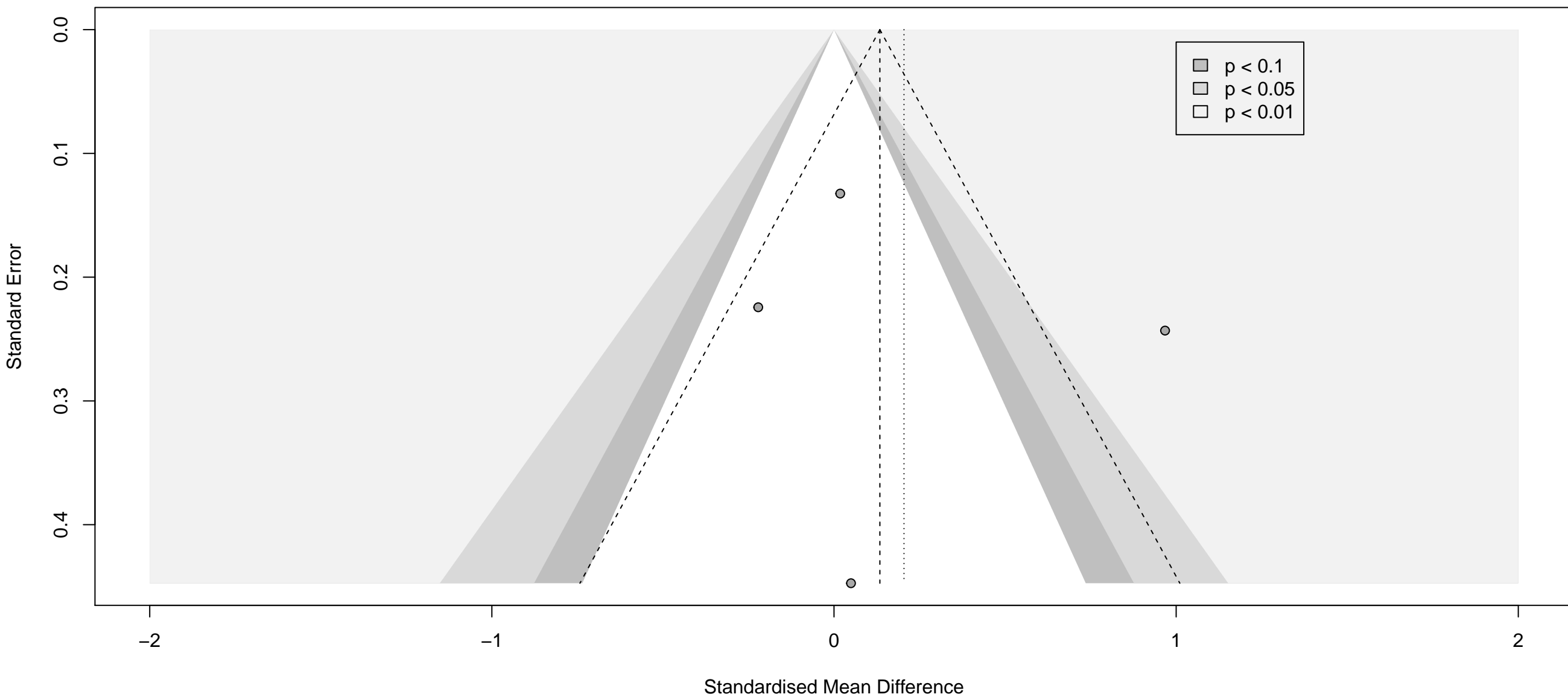

Supplement: Supplementary file 7 — Additional file 7: Figure 7. Funnel plot of the studies included in Baseline vs Day 1. [file 12967_2022_3312_MOESM7_ESM.pdf]

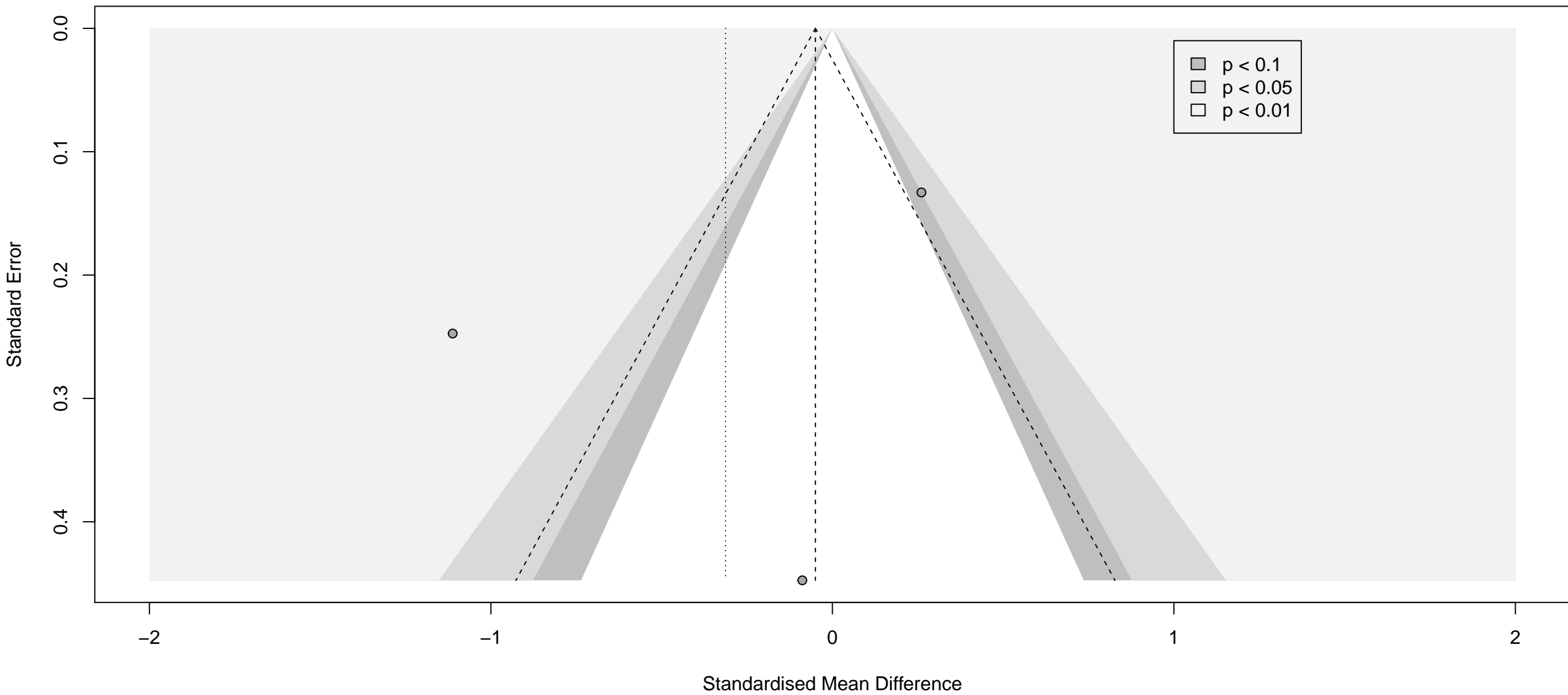

Supplement: Supplementary file 8 — Additional file 8: Figure 8. Funnel plot of the studies included in Day 1 vs Week 1. [file 12967_2022_3312_MOESM8_ESM.pdf]

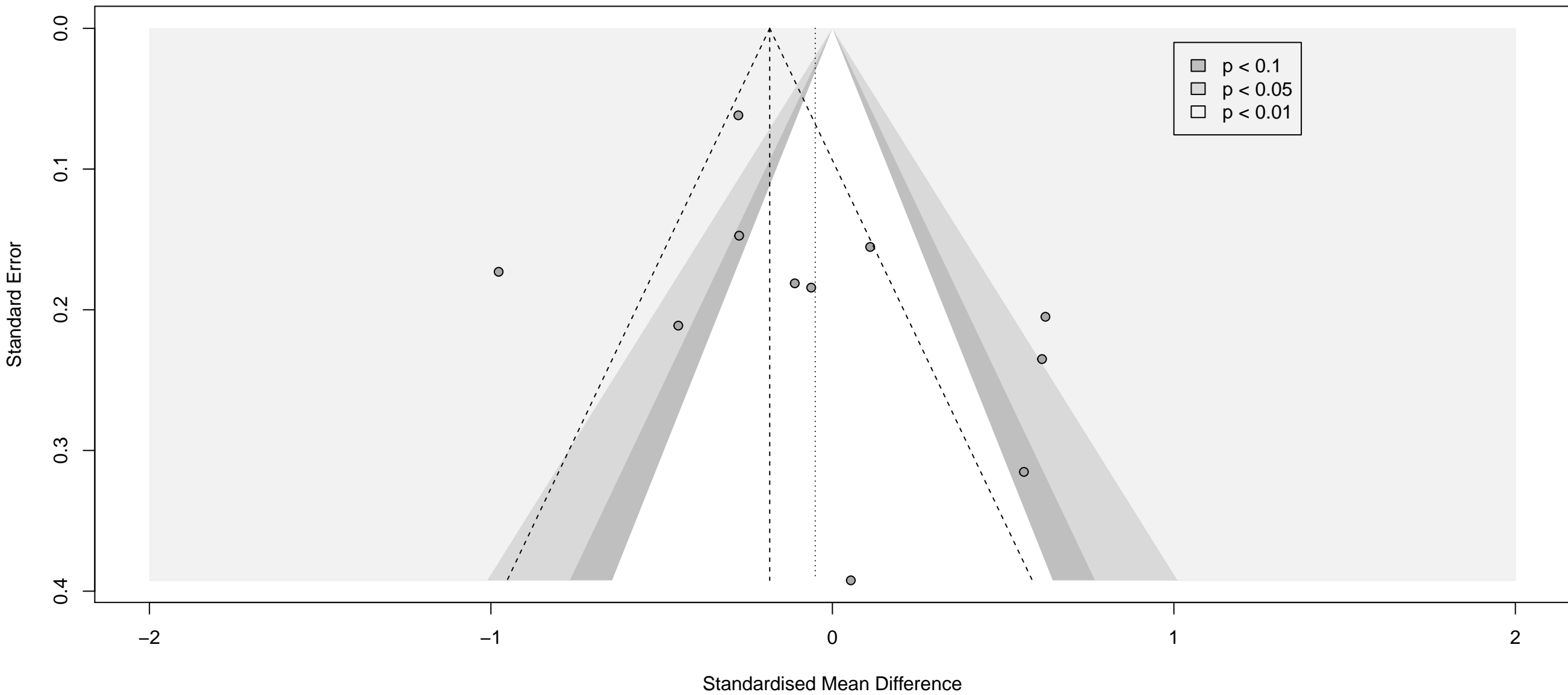

Supplement: Supplementary file 9 — Additional file 9: Figure 9. Funnel plot of the studies included in Baseline vs Over 1 month. [file 12967_2022_3312_MOESM9_ESM.pdf]

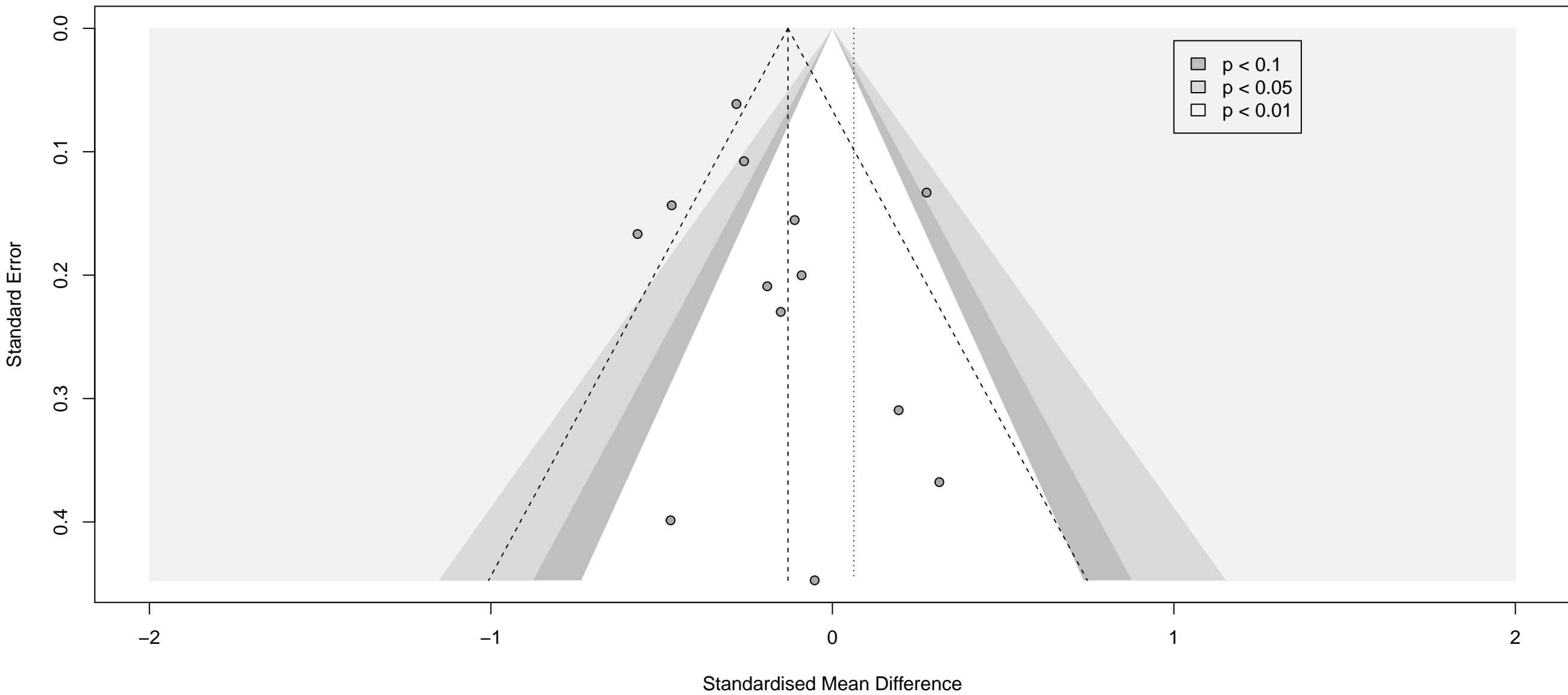

Supplement: Supplementary file 10 — Additional file 10: Figure 10. Funnel plot of the studies included in Baseline vs Week 1. [file 12967_2022_3312_MOESM10_ESM.pdf]

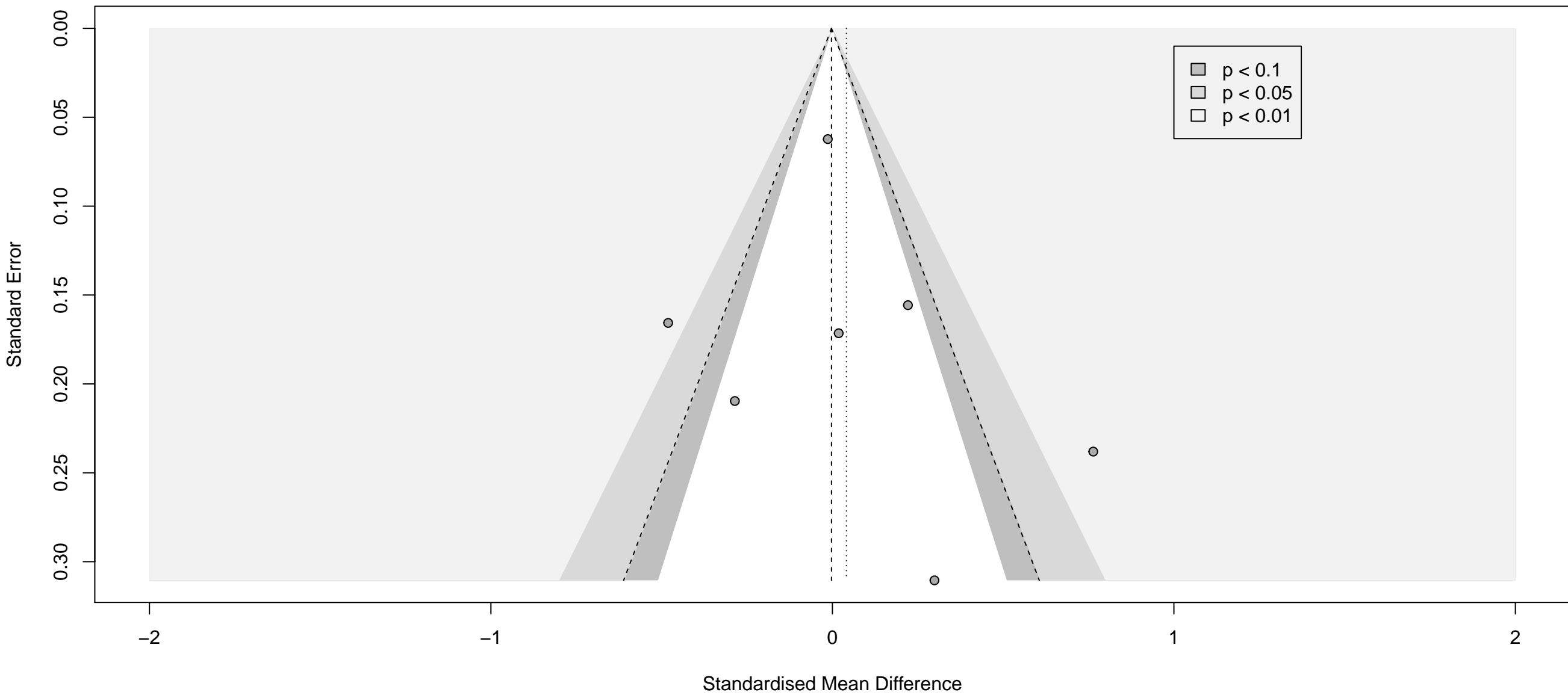

Supplement: Supplementary file 11 — Additional file 11: Figure 11. Funnel plot of the studies included in Week 1 vs Over 1 month. [file 12967_2022_3312_MOESM11_ESM.pdf]

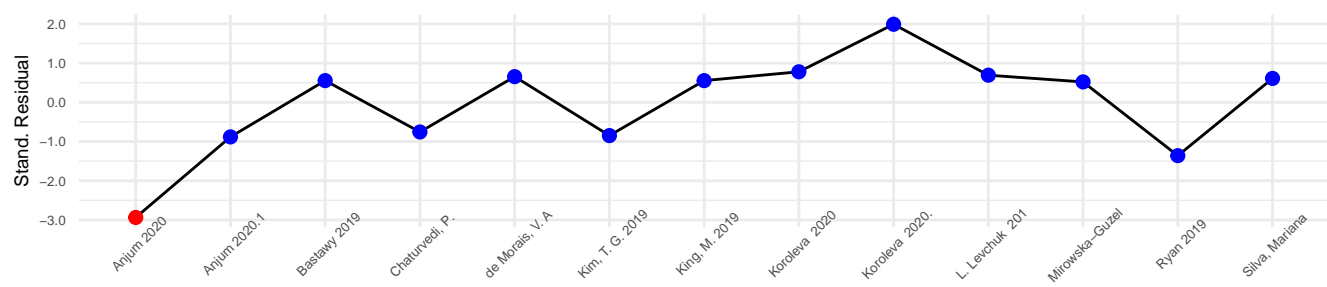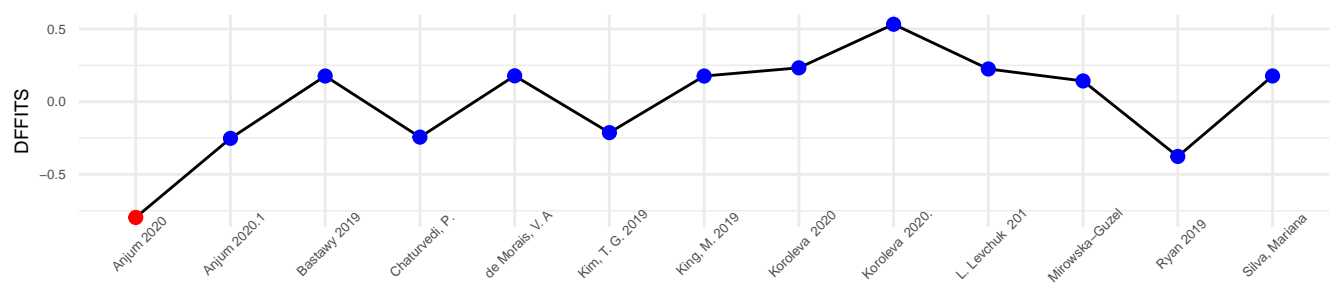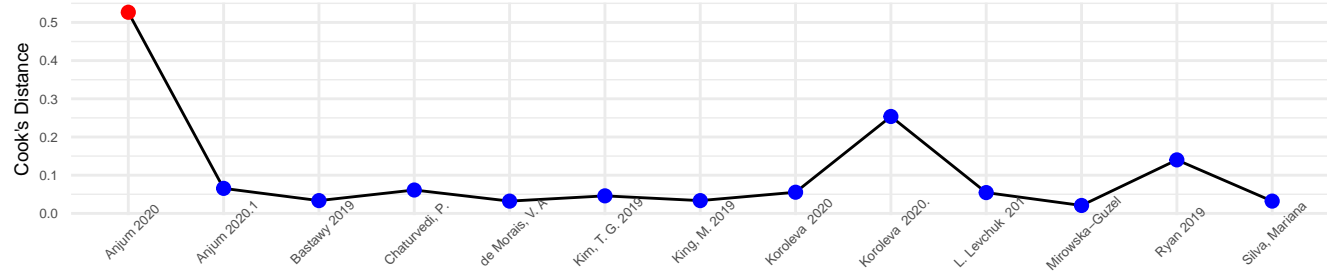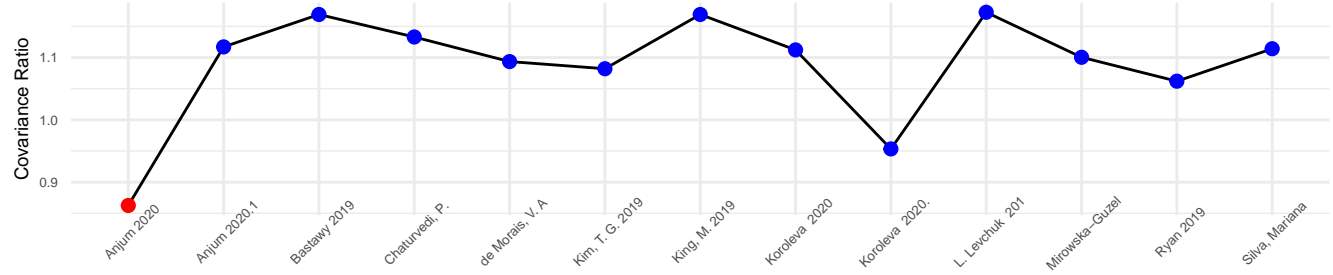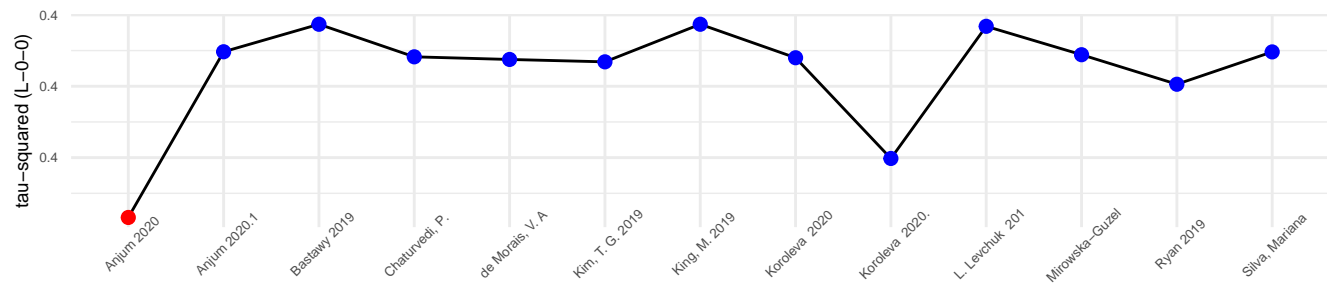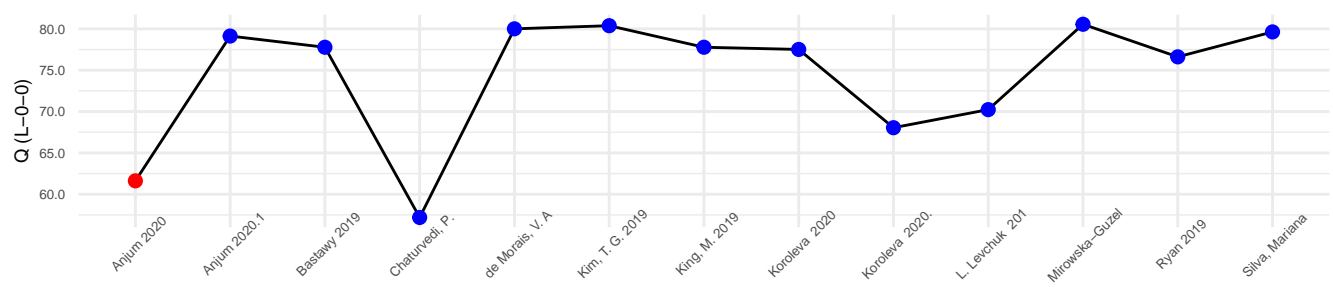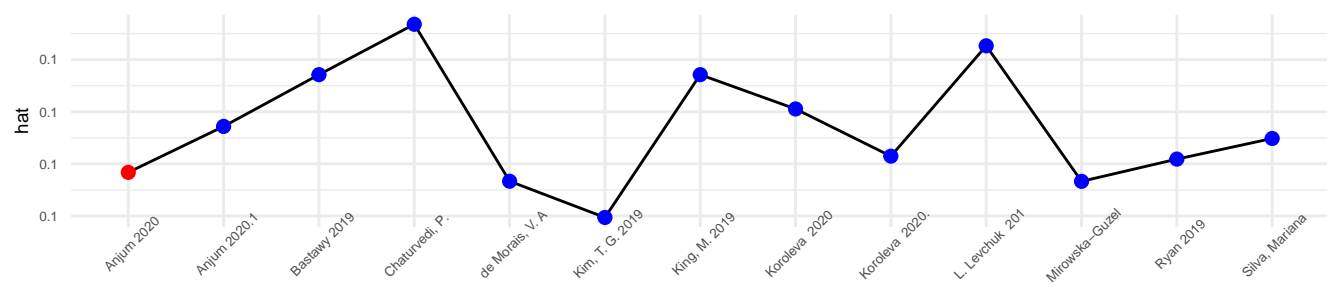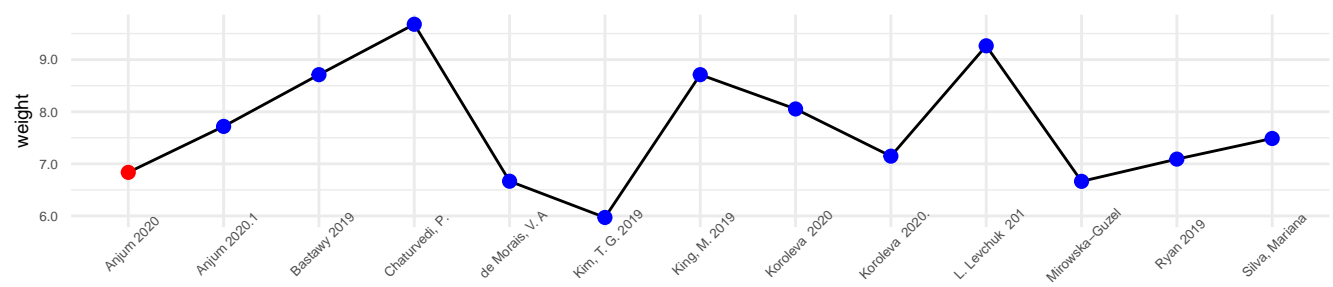

Supplement: Supplementary file 12 — Additional file 12: Figure 12. Influence analysis plot of BDNF levels in physical training subgroup baseline vs. immediate after the training. The study of Anjum et al. 2020 was found influential. [file 12967_2022_3312_MOESM12_ESM.pdf]

Sorted by Effect Size

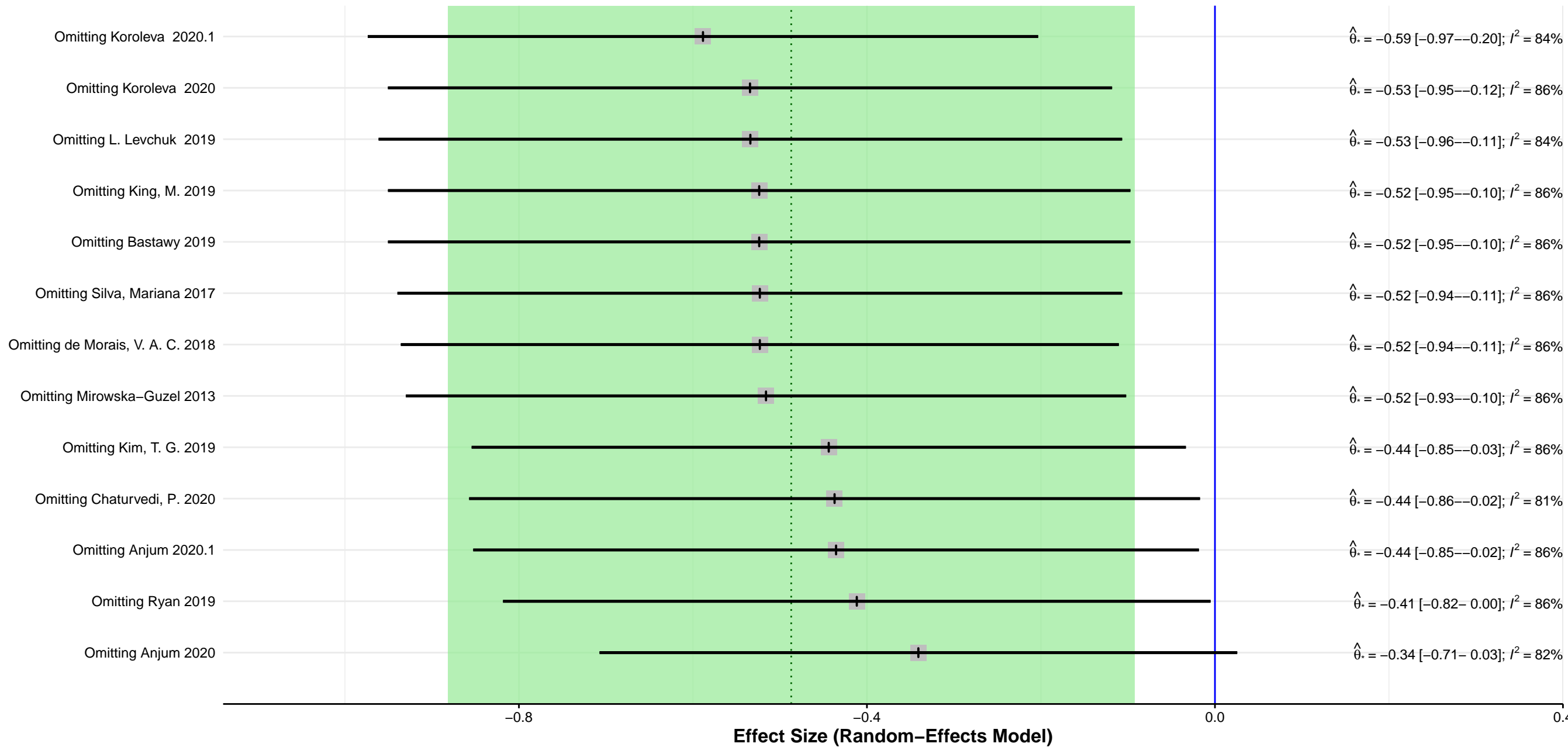

Supplement: Supplementary file 13 — Additional file 13: Figure 13. Influence analysis, ‘leave one out’ plot, of BDNF levels in physical training subgroup baseline vs. immediate after the training. Omitting the study of Anjum et al. 2020 resulted in a non-significant difference between the two groups. [file 12967_2022_3312_MOESM13_ESM.pdf]

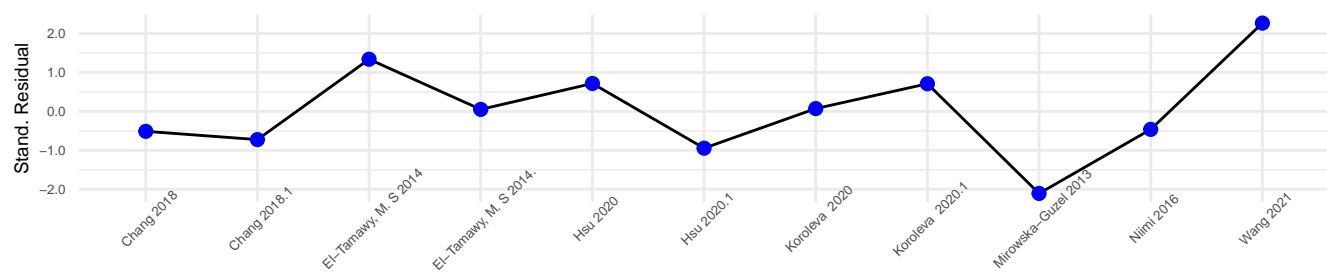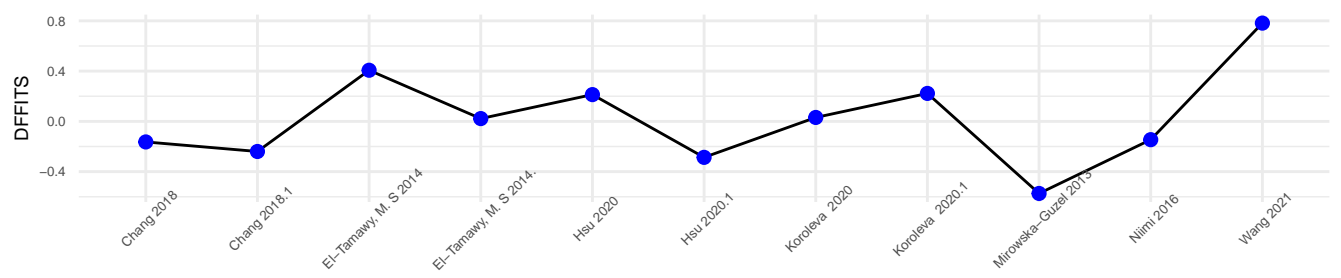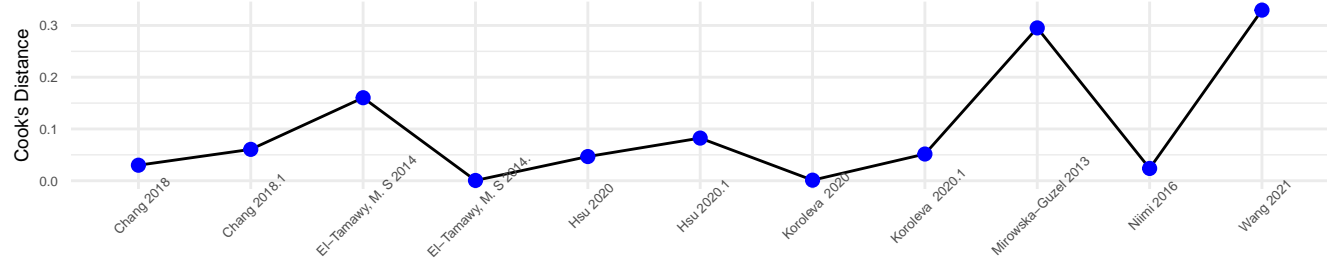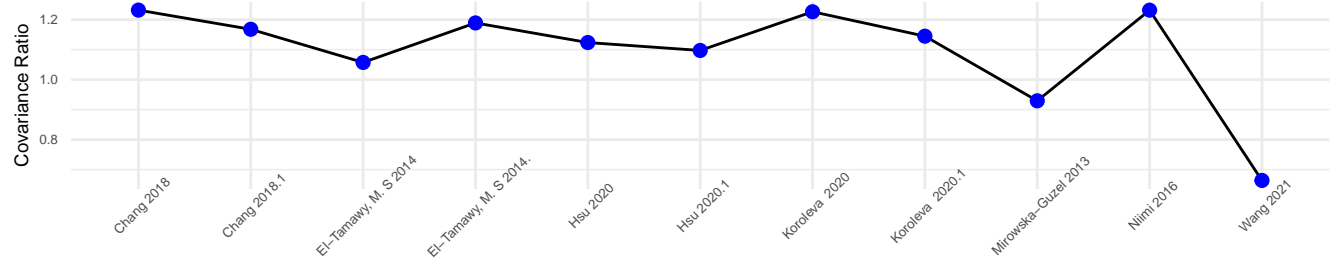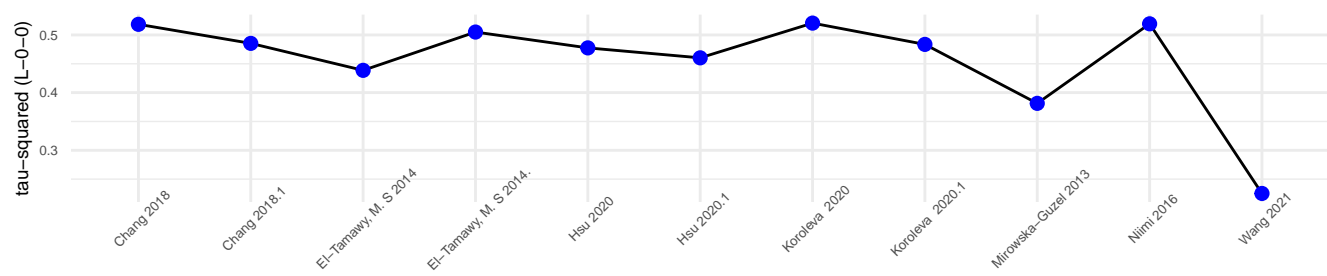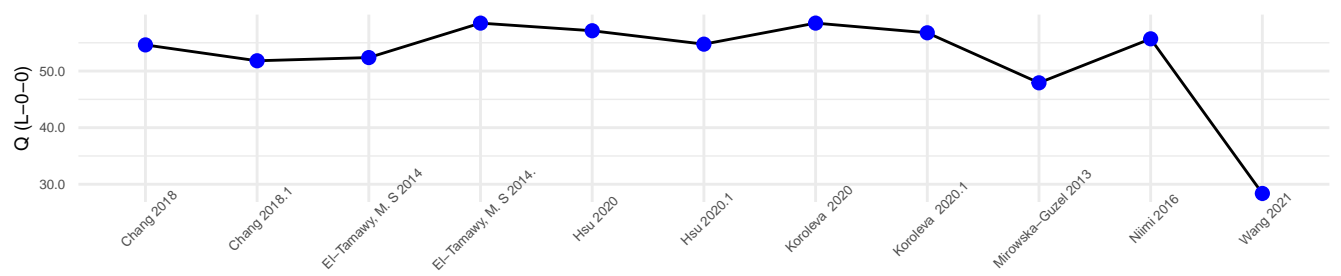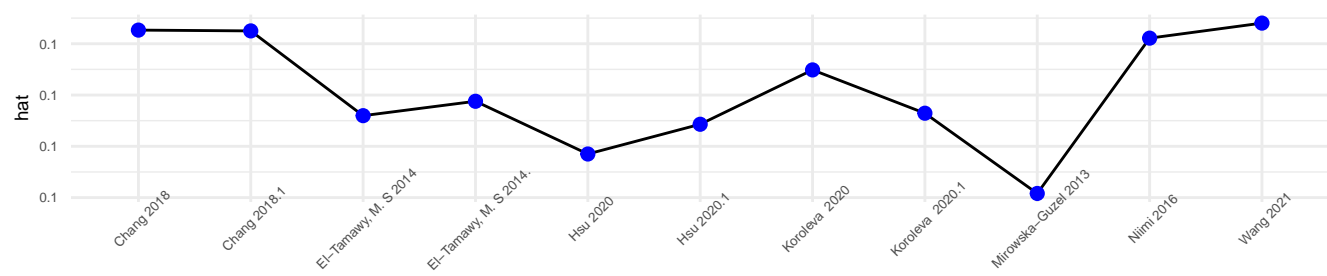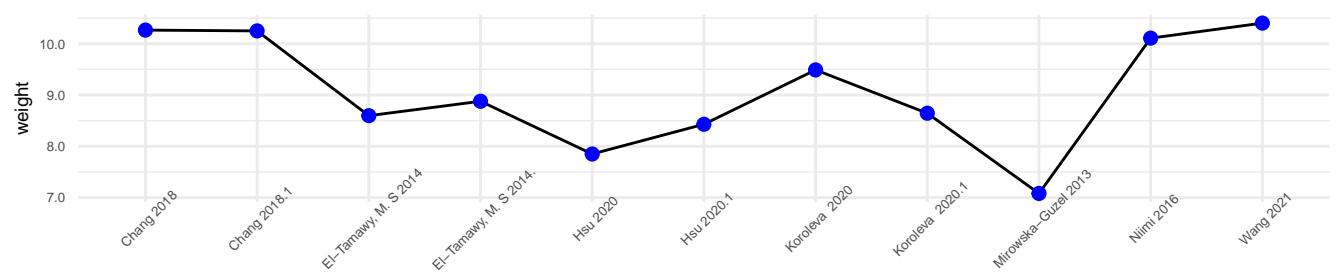

Supplement: Supplementary file 14 — Additional file 14: Figure 14. Influence analysis plot of BDNF levels in physical training subgroup baseline vs. with a delayed period after the training. No influential study was found. [file 12967_2022_3312_MOESM14_ESM.pdf]

# Sorted by Effect Size

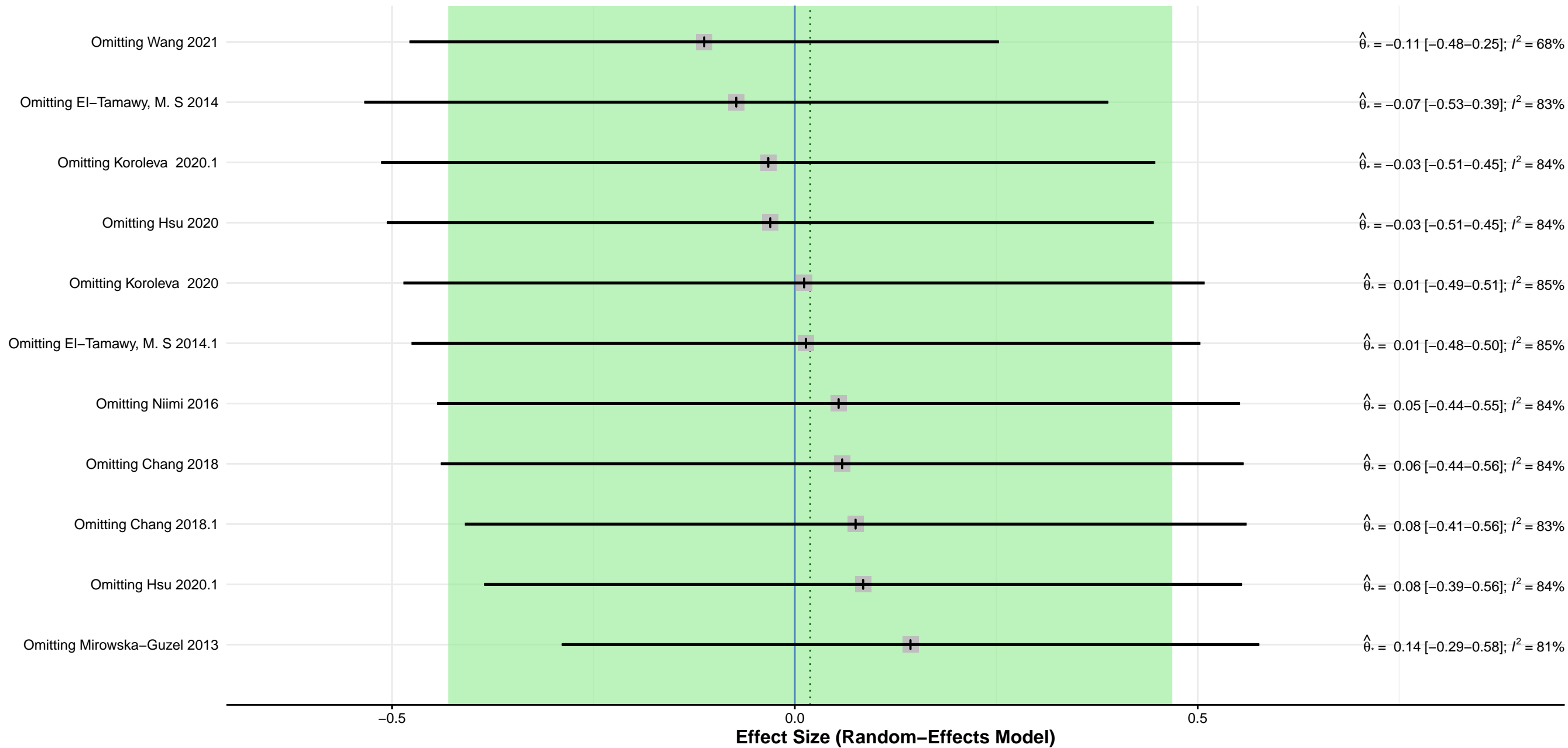

Supplement: Supplementary file 15 — Additional file 15: Figure 15. Influence analysis, ‘leave one out’ plot, of BDNF levels in physical training subgroup baseline vs. with a delayed period after the training. After omitting the study of Wang et al. 2021 the I2 index reduced to 68%. [file 12967_2022_3312_MOESM15_ESM.pdf]
