# Supplementary material for: Circulating brain-derived neurotrophic factor as a potential biomarker in stroke: a systematic review and meta-analysis
Source: J Transl Med. 2022 Mar 14;20:126. doi: 10.1186/s12967-022-03312-y (PMC8919648; doi:10.1186/s12967-022-03312-y)
Supplement: Supplementary file 2 — Additional file 2: Figure 2. Meta-analysis of the BDNF levels in PwS, Baseline vs Day 1. We found no significant difference between the two groups. [file 12967_2022_3312_MOESM2_ESM.pdf]

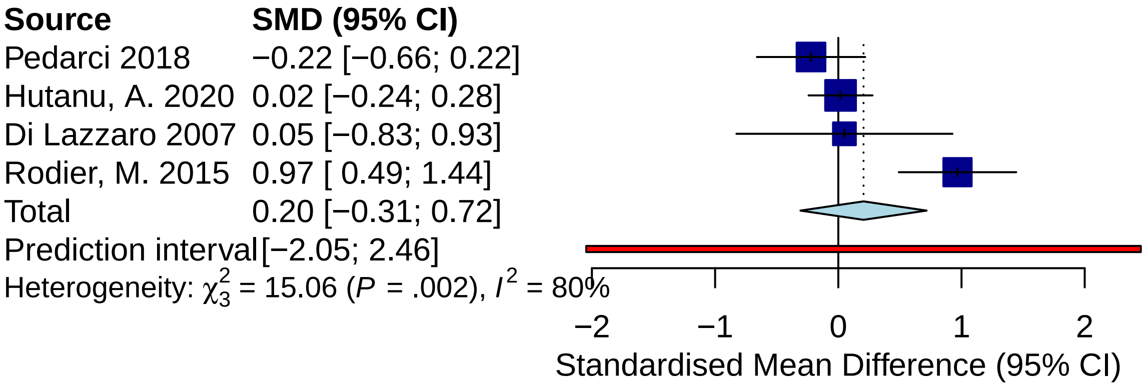
Source
SMD (95% CI)
Pedarci 2018
−0.22 [−0.66; 0.22]
Hutanu, A. 2020
0.02 [−0.24; 0.28]
Di Lazzaro 2007
0.05 [−0.83; 0.93]
Rodier, M. 2015
0.97 [0.49; 1.44]
Total
0.20 [−0.31; 0.72]
Prediction interval
[−2.05; 2.46]
Heterogeneity: χ^2_3 = 15.06 (P = .002), I^2 = 80%
−2
−1
0
1
2
Standardised Mean Difference (95% CI)
