# Supplementary material for: Circulating brain-derived neurotrophic factor as a potential biomarker in stroke: a systematic review and meta-analysis
Source: J Transl Med. 2022 Mar 14;20:126. doi: 10.1186/s12967-022-03312-y (PMC8919648; doi:10.1186/s12967-022-03312-y)
Supplement: Supplementary file 4 — Additional file 4: Figure 4. Meta-analysis of the BDNF levels in PwS, Baseline vs Over 1 month. We found no significant difference between the two groups. [file 12967_2022_3312_MOESM4_ESM.pdf]

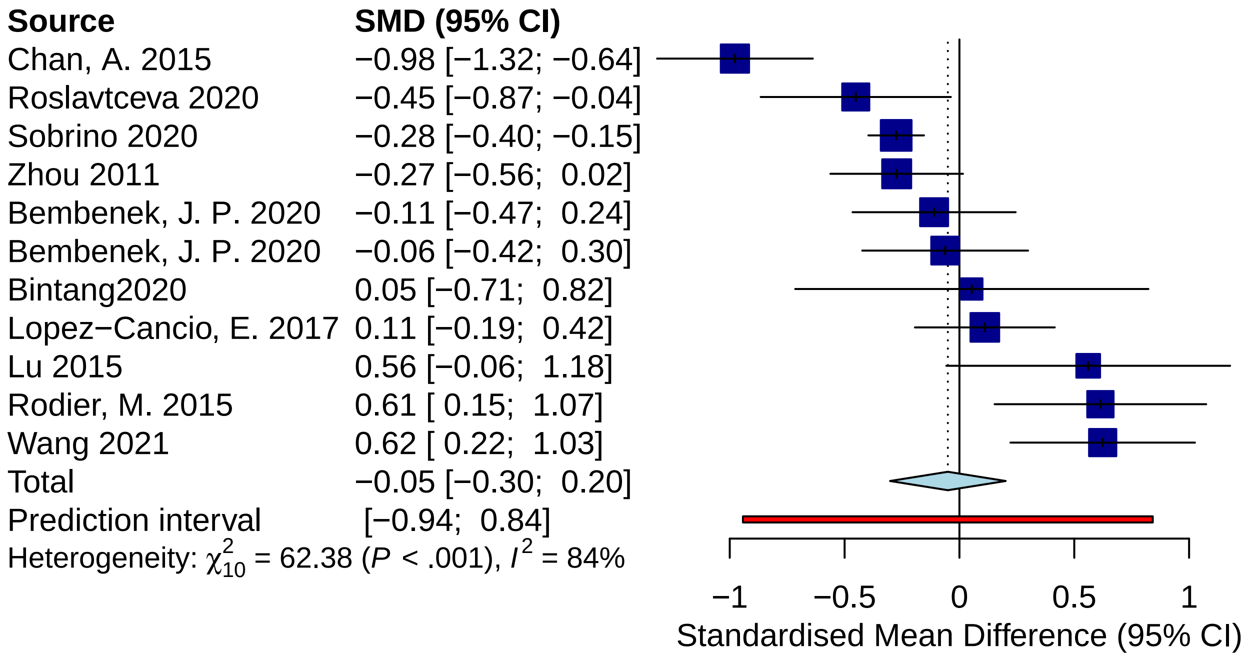
Source
SMD (95% CI)
Chan, A. 2015 −0.98 [−1.32; −0.64]
Roslavtceva 2020 −0.45 [−0.87; −0.04]
Sobrino 2020 −0.28 [−0.40; −0.15]
Zhou 2011 −0.27 [−0.56; 0.02]
Bembenek, J. P. 2020 −0.11 [−0.47; 0.24]
Bembenek, J. P. 2020 −0.06 [−0.42; 0.30]
Bintang2020 0.05 [−0.71; 0.82]
Lopez−Cancio, E. 2017 0.11 [−0.19; 0.42]
Lu 2015 0.56 [−0.06; 1.18]
Rodier, M. 2015 0.61 [0.15; 1.07]
Wang 2021 0.62 [0.22; 1.03]
Total −0.05 [−0.30; 0.20]
Prediction interval [−0.94; 0.84]
Heterogeneity: χ^2_{10} = 62.38 (P < .001), I^2 = 84%
−1
−0.5
0
0.5
1
Standardised Mean Difference (95% CI)
