# Supplementary material for: Circulating brain-derived neurotrophic factor as a potential biomarker in stroke: a systematic review and meta-analysis
Source: J Transl Med. 2022 Mar 14;20:126. doi: 10.1186/s12967-022-03312-y (PMC8919648; doi:10.1186/s12967-022-03312-y)

**Source**	**SMD (95% CI)**
Chan, A. 2015	−0.57 [−0.90; −0.24]
Billinger, S. A. 2018	−0.47 [−1.26; 0.31]
Yang 2011	−0.47 [−0.75; −0.19]
Sobrino 2020	−0.28 [−0.40; −0.16]
Ortega 2019	−0.26 [−0.47; −0.05]
Roslavtceva 2020	−0.19 [−0.60; 0.22]
Rodier, M. 2015	−0.15 [−0.60; 0.30]
Lopez−Cancio, E. 2017	−0.11 [−0.42; 0.19]
Mourao 2019	−0.09 [−0.48; 0.30]
Di Lazzaro 2007	−0.05 [−0.93; 0.82]
Lu 2015	0.19 [−0.41; 0.80]
Hutanu, A. 2020	0.28 [0.01; 0.54]
Asadollahi, M. 2019	0.31 [−0.41; 1.03]
Zhang 2017	2.83 [2.38; 3.28]
Total	0.06 [−0.28; 0.40]
Prediction interval	[−1.30; 1.42]

Heterogeneity: $\chi^2_{13} = 198.17$ ($P < .001$), $I^2 = 93\%$

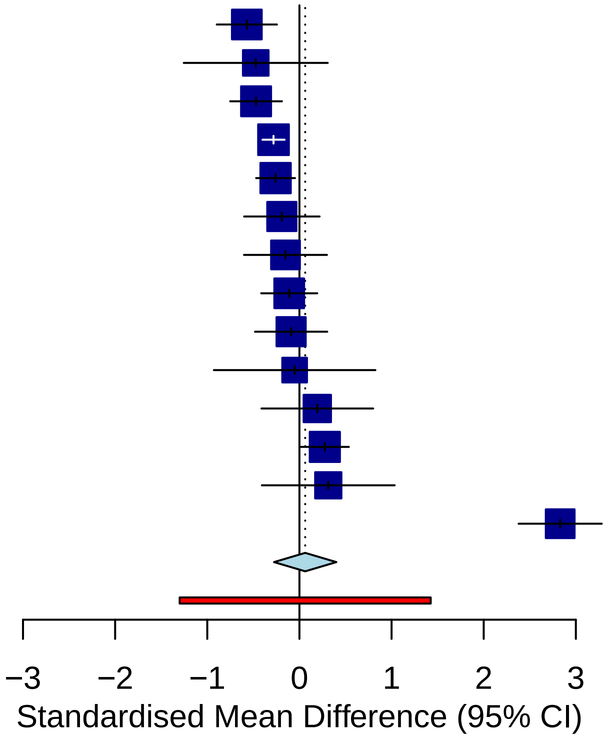

Supplement: Supplementary file 5 — Additional file 5: Figure 5. Meta-analysis of the BDNF levels in PwS, Baseline vs Week 1. We found no significant difference between the two groups. [file 12967_2022_3312_MOESM5_ESM.pdf]
