# Supplementary material for: Circulating brain-derived neurotrophic factor as a potential biomarker in stroke: a systematic review and meta-analysis
Source: J Transl Med. 2022 Mar 14;20:126. doi: 10.1186/s12967-022-03312-y (PMC8919648; doi:10.1186/s12967-022-03312-y)
Supplement: Supplementary file 6 — Additional file 6: Figure 6. Meta-analysis of the BDNF levels in PwS, Week 1 vs Over 1 month. We found no significant difference between the two groups. [file 12967_2022_3312_MOESM6_ESM.pdf]

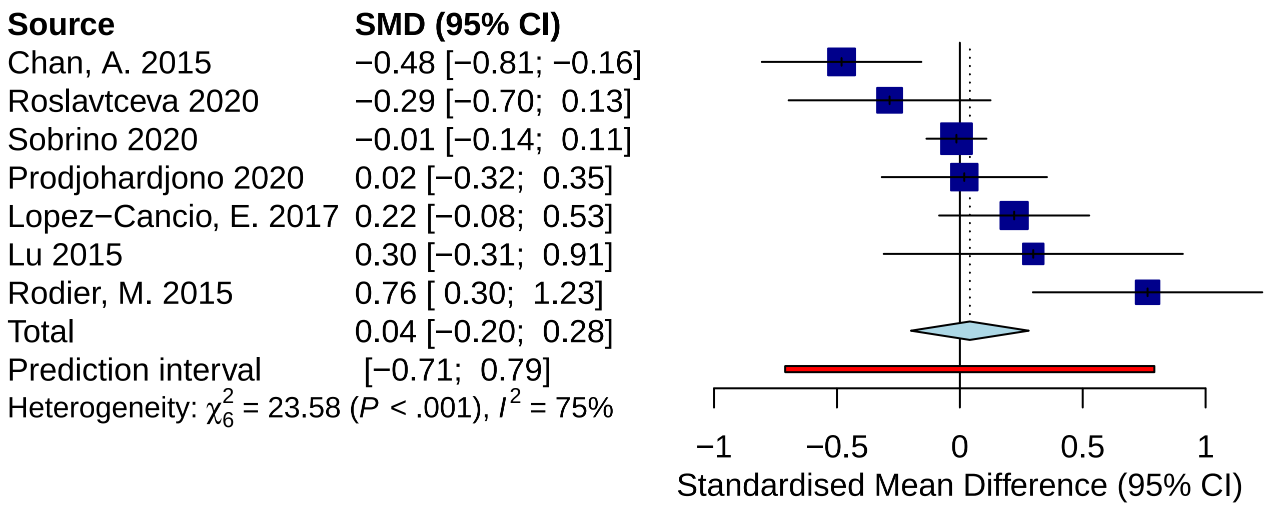
Source
SMD (95% CI)
Chan, A. 2015 −0.48 [−0.81; −0.16]
Roslavtceva 2020 −0.29 [−0.70; 0.13]
Sobrino 2020 −0.01 [−0.14; 0.11]
Prodjohardjono 2020 0.02 [−0.32; 0.35]
Lopez−Cancio, E. 2017 0.22 [−0.08; 0.53]
Lu 2015 0.30 [−0.31; 0.91]
Rodier, M. 2015 0.76 [0.30; 1.23]
Total 0.04 [−0.20; 0.28]
Prediction interval [−0.71; 0.79]
Heterogeneity: χ^2_6 = 23.58 (P < .001), I^2 = 75%
−1
−0.5
0
0.5
1
Standardised Mean Difference (95% CI)
